# An organotypic ectocervix mucosa model to study host-pathogen interaction of sexually transmitted infections

**DOI:** 10.1016/j.mtbio.2026.103454

**Published:** 2026-07-14

**Authors:** Helene Mehling, Claire Rousseau, Tobias Krammer, Alexander M. Leipold, Aziza Boyny, Saskia-Laureen Herbert, Christine Wulff, Antoine-Emmanuel Saliba, Lars Dölken, Florian Groeber-Becker, Thomas Rudel, David Komla Kessie

**Affiliations:** a Department of Microbiology, Biozentrum Am Hubland, University of Wuerzburg, Wuerzburg, Germany; b Translational Center for Regenerative Therapies, Fraunhofer Institute for Silicate Research (ISC), Wuerzburg, Germany; c Institute for Virology and Immunology, University Hospital of Wuerzburg, Wuerzburg, Germany; d Helmholtz Institute for RNA-based Infection Research (HIRI), Helmholtz Centre for Infection Research, University of Wuerzburg, Germany; e Institute for Molecular Infection Biology, University Hospital of Wuerzburg, Wuerzburg, Germany; f Institute of Experimental Ophthamology, University Hospital of Duesseldorf, Germany; g Institute of Virology, Hannover Medical School, Hannover, Germany; h Department for Obstetrics and Gynecology, University Hospital of Wuerzburg, Wuerzburg, Germany

**Keywords:** *Neisseria*, *Chlamydia*, Cervix, Sexually transmitted infection, Organotypic, Organ-on-chip

## Abstract

Organotypic models closely recapitulate *in vivo* human tissues and organs and thus overcome limitations of animal and conventional cell cultures in studying human specific infections. Infections of the female reproductive tract by sexually transmitted pathogens typically start in the lower female reproductive tract, including the vagina and ectocervix, before ascending to the upper reproductive tract. These lower reproductive tract infections are often asymptomatic, facilitating silent transmission between partners and result in severe complications due to delayed diagnosis and treatment. However, due to the lack of relevant models and the challenge of detecting clinically asymptomatic infections, research has largely focused on symptomatic infections of the upper female reproductive tract (FRT). We present an organotypic human ectocervix mucosal (hEcCxM) model, generated by coculturing primary ectocervix epithelial cells and fibroblasts on a plastically compressed collagen scaffold. Histological analysis and single-cell RNA sequencing (scRNA-seq) revealed that the hEcCxM model developed a stratified squamous epithelium and exhibited key features essential for its physiological barrier function. Transcriptional fidelity of the hEcCxM models was validated against native ectocervix tissues. The model recapitulated several *in vivo* infection phenotypes of *Neisseria gonorrhoeae* (*GC*) and *Chlamydia trachomatis* (*Ctr*)*.GC* infection induced secretion of proinflammatory cytokines while Ctr infections did not elicit a comparable inflammatory response. Additionally, we demonstrated that neutrophils could migrate to infection sites from the basal side, but this requires the presence of an endothelial layer. Overall, the hEcCxM model represents a scalable and physiological platform for studying host-pathogen interactions in sexually transmitted infections, as well as non-infectious diseases affecting the ectocervical mucosa.

## Graphical abstract

**Figure:**
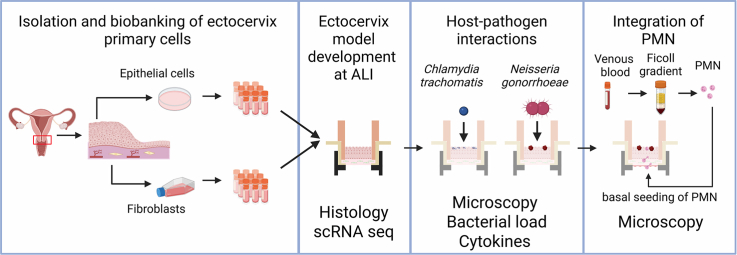

## 1 Introduction

The cervix forms the boundary between the lower and upper female reproductive tract (FRT) and is anatomically divided into the ectocervix (EcCx) and endocervix, which is connected by a transition zone. The EcCx protrudes into the vagina to form the cervico-vaginal orifice, while the endocervix lines the uterocervical canal. Structurally, the EcCx is a uniform stratified, non-keratinizing squamous epithelium whereas the endocervix consists of a fissured, mucus producing monolayered epithelium. The cervical wall contains smooth muscle interspersed with abundant collagen-rich connective tissue [1]. A basement membrane separates the EcCx epithelium from the underlying connective tissue and supports basal stem cells. These basal cells are polygonal with large nuclei capable of dividing and differentiating to generate the upper cell layers, which are organized into parabasal, intermediate and superficial layers. During EcCx cell maturation, their cell body increases in size while the nuclear size decreases. Additionally, the intermediate and superficial cells contain glycogen. The uppermost layer of the EcCx mucosa is flattened, lacks microvilli and forms a protective barrier that is continuously shed and replaced by the underlying suprabasal layer. The distribution of junctional proteins such as ZO-1 and E-cadherin within the *ex vivo* ectocervix mucosa is mainly restricted to the lower two-thirds of the tissue, where they exhibit characteristic “spider web” pattern between the cells. In contrast, the apical layers show reduced expression of junctional proteins facilitating the diffusion of signaling molecules to the lower layer [2]. The cervix separates the upper reproductive tract from the lower reproductive tract and is the primary entry site for many sexually transmitted pathogens. Infection of the lower reproductive tract by sexually transmitted pathogens such as *Chlamydia* and *Neisseria* can remain clinically asymptomatic for several weeks to months and result in the silent spread of the pathogens among sex partners [3,4].

*Chlamydia trachomatis* (*Ctr*) and *Neisseria gonorrhoeae* (*GC*) are the two most common sexually transmitted bacterial pathogens worldwide [5]. Infection by both pathogens typically begins at the mucosal surfaces, the urethra in males and the cervix in females, but they can also infect anal, pharyngeal and ocular epithelia [[6], [7], [8]]. Women are generally more susceptible to infections by both pathogens [7]. In women, these pathogens first infect the EcCx before they spread into the upper reproductive tract via currently unknown mechanisms although peristaltic movement and neutrophil involvement have been suggested [9]. If left untreated, infections can lead to serious complications such as pelvic inflammatory disease, epididymitis, infertility and ectopic pregnancy [[10], [11], [12], [13]]. *Chlamydia* are obligate intracellular bacteria with a biphasic life cycle consisting of infectious elementary bodies (EB) and replicative reticulate bodies (RB). EBs adhere to epithelial cells via surface receptors upon contact and enter the host cells enclosed in a membrane-bound compartment, the so-called inclusion. In the inclusion, the EBs differentiate into the metabolically active RB, which proliferate and then re-differentiate into EBs. These newly formed EBs are then released in vesicles by extrusion or after cell lysis to start a new infection cycle [14,15]. In contrast, *GC* is a gram-negative pathogen that adheres to and invades susceptible cells, primarily through type IV pili and opacity proteins [[16], [17], [18]]. *Ctr* and *GC* infection are highly human specific pathogens and have therefore developed multiple ways to evade the host immune defenses [[19], [20], [21], [22], [23], [24], [25]]. Infection by either pathogen induces the influx of neutrophils into the tissue causing inflammation and the clinical symptoms associated with these infections [10,17,[26], [27], [28]].

Animal models and conventional two-dimensional (2D) epithelial cell cultures have been essential in advancing our understanding of several aspects of host-pathogen interactions, but they are limited in replicating the in-situ complexity of human infections. Conventional 2D cultures lack the cell-cell and cell-tissue contacts of native tissues, while murine models rapidly clear infection and require use of mouse-adapted strains, hormonal treatments or transcervical infections to establish disease [29]. Tissue explants and organoids offer more physiological conditions that better capture tissue complexity. However, tissue explants are not scalable and are limited by material availability while infections in organoids often do not fully mimic physiological conditions [[30], [31], [32]]. Tissue engineering has enabled the development of three dimensional (3D) *in vitro* organotypic models that closely recapitulate the morphological and physiological properties of tissues *in vivo* [26,[33], [34], [35], [36], [37], [38]] to overcome many limitations of traditional models.

We have developed a complex immunocompetent *in vitro* human EcCx mucosal (hEcCxM) model by co-culturing primary ectocervix fibroblasts and epithelial cells on a plastically compressed collagen gel. In this study, we used the hEcCxM models to assess the infection dynamics of *GC* and *Ctr* and the recruitment of neutrophils to the sites of infection. Our hEcCxM models developed a stratified squamous epithelium and expressed receptors typical of the *in vivo* tissue. *GC* and *Ctr* successfully colonized the tissue models and induced the influx of neutrophil from the basal side to the site of infection, closely mimicking physiological host-pathogen interactions.

## 2 Materials and methods

### 2.1 Ethical statement

This study was approved by the University Hospital of Wuerzburg Ethical Committee (Votes: 37/16, 36/16, 261/20). Usage of venous blood and neutrophils from healthy donors was approved by University of Wuerzburg Ethics Commission (Vote: 300/21). All donors signed an informed consent form.

### 2.2 Isolation of cervix fibroblasts and epithelial cells

**Fig. 1:**
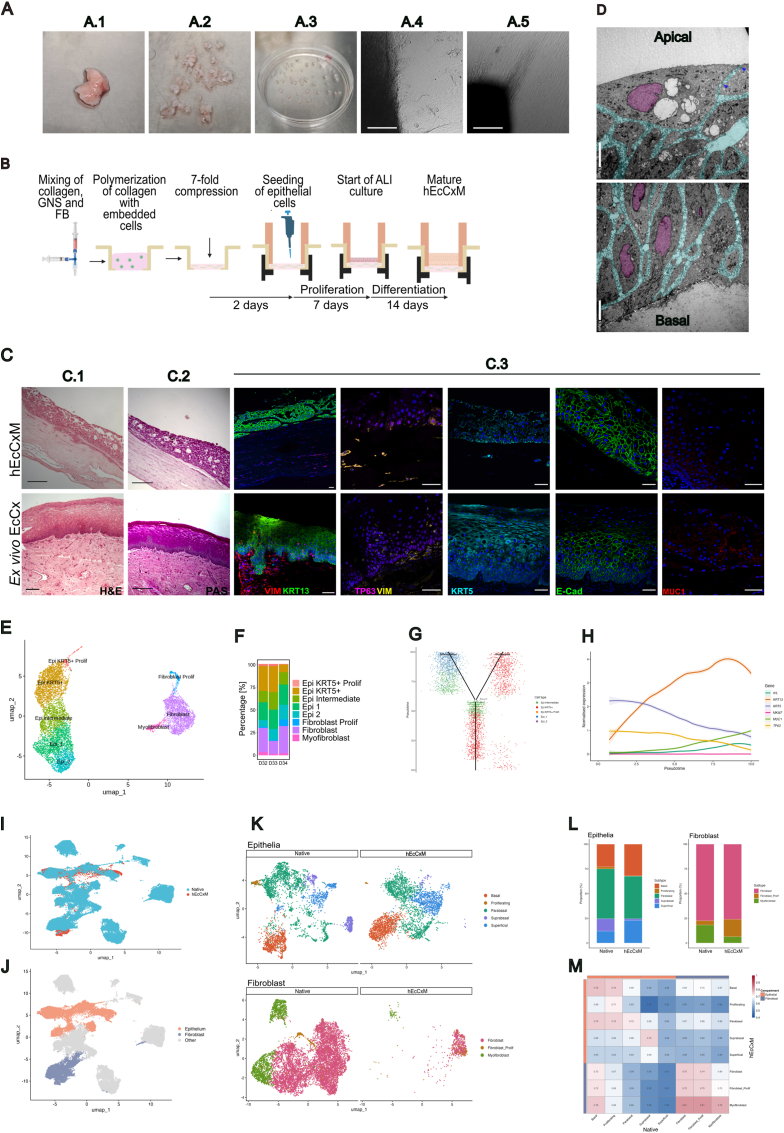
**Development and characterization of *in vitro* hEcCxM models. A:** Cell isolation from EcCx biopsy. Ectocervix mucosa after dissecting connective tissue(A.1), mucosa pieces after mincing with scalpel (A.2), adhered mucosal pieces in 10 cm plate after overnight culture(A.3) and brightfield microscope images showing outgrowth of epithelial cells (A.4) and fibroblasts (A.5) from mucosal pieces (Scale bar: 200 μm). **B:** Schematic of development of hEcCxM models (Created with www.biorender.com). **C:** Representative images of histological staining with haematoxylin-eosin (C.1), Periodic-Acid-Schiff(C.2) and immunohistological characterization (C.3) of hEcCxM models (Scale bar: 50 μm). **D:** TEM images (colorized in Inkscape) showing flattened apical cells (top) and polygonal-shaped basal cells (bottom) of the epithelial layer of the hEcCxM models. Nucleus is colored magenta and intercellular space is colored cyan with finger-like interconnections between adjacent cells (Scale bar: 5 μm). **E:** UMAP representation of single-cells color coded by cell types **F:** Relative proportion of the cell types identified from hEcCxM models developed from three different donors. **G:** URD differentiation tree of hEcCxM model epithelium showing pseudotime values from early(bottom) to late (top). Each dot represents a single cell colored by subcluster. H: Expression dynamics of selected ectocervix genes along pseudotime. **I:** UMAP showing clustering of hEcCxM models and native tissues after data integration. **J:** UMAP highlighting epithelia and fibroblast clusters from other cell types after data integration. **K:** UMAP projection of epithelia and fibroblast from native tissues and hEcCxM model showing annotated subtypes based on transcriptional identity. **L:** Bar graph showing proportions of epithelial cells and fibroblasts from native and hEcCxM after integration. **M:** Heatmap of Spearman ranked correlation coefficient matrix of epithelia and fibroblast subclusters between native tissues and hEcCxM after integration. (For interpretation of the references to color in this figure legend, the reader is referred to the Web version of this article.)

Primary epithelial cells and fibroblasts were isolated from donor materials from patients undergoing hysterectomies at the University Hospital of Wuerzburg after informed consent. Cells were isolated as previously described by Steinke et al. [35] from tracheobronchial tissues and summarized in Fig. 1A. Briefly, to isolate epithelial cells, the connective tissue layer was trimmed as much as possible using sterile forceps and scalpel (Fig. 1A1). The mucosal layer was then chopped into tiny pieces (Fig. 1A2) and placed with the epithelial side facing downwards in a 7,5 μg/cm^2^ collagen-coated (FisherScientific, 11519816) 10 cm cell culture dish (Sarstedt, 83.3902). The pieces were covered with 1 ml ectocervix epithelial cell growth medium (EcCxGM) adapted from Chumduri et al. [39] (Table S 1) and cultured overnight to allow the tissues to adhere to the plate (Fig. 1A3). 5 ml of EcCxGM was added to the tissue pieces the following day and incubated for a further 6 days. Epithelial cells were observed to grow out on the third day (Fig. 1A4). The tissue pieces were transferred after 7 days of culture into fresh collagen-coated plates, and the process was repeated 3-5 times. To isolate patient-matched fibroblasts, the connective tissue was minced into tiny pieces in 10 cm cell culture plates with sterile scalpel. The pieces were covered with 1 ml fibroblast growth medium (FGM) consisting of Dulbecco's Modified Eagles Medium (Merck/Sigma Aldrich, D6429) supplemented with 10% fetal bovine serum (FCS) (ThermoFisher, A5256701) and cultured overnight. 6 ml FGM was added the next day and the tissue pieces cultured until cells were observed to grow out between 5 and 7 days (Fig. 1A5). The cells were expanded for another 5 days before freezing. The isolated cells were detached with TrypLE (ThermoFisher, 12604039) and 500,000 cells per vial were frozen in Cryo-SFM Plus freezing medium (Promocell, C-29922) supplemented with 10% FCS. All the media for cell isolation and expansion contained 0.1 mg/ml streptomycin, 100 U/ml penicillin (Merck/Sigma Aldrich, P4333) and 0.25 μg/ml Amphotericin B (Merck/Sigma Aldrich, A2942). Isolated cells were tested by PCR for mycoplasma (Primer: GPO: 5′ ACT CCT ACG GGA GGC AGC AGTA 3′, MGSO: 5′ TGC ACC ATC TGT CAC TCT GTT AAC CT 3′, HGH I: 5′ TTC CCA ACC ATT CCC TTA TCC AGG 3′, HGH II: 5′ TCC ACT CAC GGA TTT CTG TTG TGT TT 3′). All cell culture and infection incubation were done in a humidified incubator at 37°C and 5% CO_2_, unless otherwise stated.

### 2.3 Cell culture and development of the human ectocervical mucosa model

Isolated ectocervix (EcCx) epithelial cells were expanded by seeding them on mitomycin C (bio-techne, 3258/10) treated mouse embryonic feeder cells with ectocervix epithelial cell growth medium (EcCxGM). Fibroblasts were maintained in fibroblast growth medium (FGM) until 80-90% confluency. The *in vitro* hEcCxM models were developed on plastically compressed collagen gels as previously described by Reuter et al. [40,41] using either 7 mm or 12 mm custom made inserts, so-called cell crowns (Figure S 3A). Briefly, 2 parts high concentration rat tail collagen (8-12 mg/ml) was mixed with 1 part gel neutralizing solution (GNS) containing the fibroblasts to a final concentration of 40,9 μg/mm^2^ collagen and 260 fibroblasts/mm^2^ using two 20 ml syringes (FisherScientific, 10310064) and a 3-way-valve (FisherScientific, 10136353). For a 10 mg/ml collagen, 750 μl (12 mm) or 225 μl (7 mm) of the mixture was pipetted into the compression chamber with a multistep pipette (Brand, 705110). The collagen was polymerized by incubating at 37°C and 5% CO_2_ for 1 h. The polymerized collagen was then subjected to a 7-fold compression at room temperature (RT) with weights to reach a thickness of 1 mm for 1 h (Fig. 1B). The cell crowns were assembled (Figure S 3B,C) and placed in either 6-well (12 mm) or 12-well (7 mm) plates with 4 or 2 ml FGM in the basal compartment and 300 or 100 μl in the apical and incubated overnight. The ectocervix epithelial cells were detached by incubating with Versene (ThermoFisher, 15040066) for 4 min and then TrypLE for another 4 min. 2500 epitheilal cells/mm^2^ were seeded in the apical compartment in 100 μl EcCxGM. The basal medium was changed to a 1:1 mix of EcCxGM:FGM (co-culture medium). The models were cultured under submerged conditions for 7 days and subsequently airlifted for another 14 days with either 2 ml (12 mm) or 1 ml (7 mm) co-culture medium in the basal compartment (Figure S 3D). The medium in the basal compartment was changed every second day.

### 2.4 Characterization of the human ectocervical mucosa models

For histological analysis, the human ectocervix mucosal (hEcCxM) models were fixed with 4% paraformaldehyde (PFA) (Morphisto, 11762.01000) overnight at 4°C, dehydrated in an ascending ethanol series, followed by immersion in isopropanol and acetone for 1 h each, paraffinization in a TP1020 tissue processor (Leica Biosystems) and embedded in paraffin. 6 μm sections of the formalin-fixed paraffin embedded (FFPE) tissues were then cut on a Microm HM315 microtome (Leica Biosystems), fixed on frosted slides (Hartenstein, #OTS), deparaffinated and rehydrated by a series of alcohol concentrations and stained with Haematoxylin-Eosin (H&E) and Periodic-Acid-Schiff (PAS). For immunofluorescence staining, the FFPE sections were deparaffinated and rehydrated, demasked for 20 min in 1x citrate buffer (pH6) or Ethylenediaminetetraacetic acid buffer (EDTA, pH9) at 100°C and blocked with 3% bovine serum albumin (BSA)/1x phosphate-buffered saline (PBS) (Roth, 8076.3) at room temperature for 1 h. The sections were then covered with primary antibodies (Table S 2) diluted in 1% BSA(1x PBS) and incubated at 4°C overnight. The sections were washed 3 × 5 min with washing buffer (1x PBS/0.5% Tween 20 (Roth, 9127.2)) and counterstained with fluorescently conjugated secondary antibodies (Table S 2) for 1 h at RT. The sections were washed again 3x for 5 min with washing buffer and mounted with Fluoromount-G with DAPI (ThermoFisher, 00-4958-02). Slides were airdried overnight at RT in the dark prior to imaging.

### 2.5 Wholemount staining and confocal microscopy

The tissue models were washed 3x with sterile Dulbecco's PBS (DPBS) (ThermoFisher, 14190169) and fixed with 4% PFA overnight at 4°C. The 4% PFA was removed and the tissues washed with PBS. The models were then detached from the cell crowns and placed in a 96-well plate (7 mm) or 24 well plate (12 mm). The tissues were blocked and permeabilized with 3% BSA/PBS with 0.1% tritonX100 (Roth, 3051.4) for 1 h at RT. The blocking solution was removed, and the tissues were then covered with primary antibody (Table S 2) diluted with 1% BSA/PBS with 0.01% TritonX-100 and incubated overnight at 4°C. The models were then washed 3x with washing buffer for 5 min and counterstained with fluorophore-conjugated secondary antibodies (Table S 2) and DAPI (Sigma Aldrich, D9542-5 MG) at RT for 1 h. The models were washed again and mounted with a drop of ProLong Glass Antifade mounting solution (ThermoFisher, P36983) with silicon spacers (ThermoFisher, P36983) on two coverslips (Hartenstein, DK60). The mounted models were kept at RT overnight in the dark to polymerize the mounting medium. A Z-stack through approximately 100 μm from 3 independent experiments was captured on either Leica Stellaris 5 or SP5 (Leica Microsystems) and processed with FIJI [42] or IMARIS v 8.4.1.

### 2.6 Electron microscopy

Ultrastructural analysis of the hEcCxM models was performed at the imaging core facility of the University of Wuerzburg. For transmission electron microcopy (TEM), the tissues were fixed with 2.5% cacodylate-buffered glutaraldehyde (50 mM cacodylate, pH 7.2, 50 mM KCl and 2.5% MgCl_2_) at 4°C overnight. Tissues for scanning electron microscopy (SEM) were fixed with 6.5% glutaraldehyde. Further processing of the tissues proceeded as described by Spiliotis et al. [43]. SEM images were made with the JEOL JSM-7500F and TEM images with JEOL JEM-1400 Flash.

### 2.7 Polymorphonuclear neutrophil (PMN) migration assays

To add polymorphonuclear neutrophils (PMNs) to the models, human umbilical vein endothelial cells (HUVECs) were first seeded to the base of the tissue on the 14th day of development. The model was flipped with the basal side up, pretreated with 5 μg/cm^2^ fibronectin (Roche, 10838039001) and incubated for 15 min before seeding 2500 HUVECs/mm^2^ to the base of the models in VascuLife complete medium (CELLSYSTEMS, LL-0003). The model was incubated for 4 h before being reflipped (apical up). The medium in the basal compartment was changed to 1:1:1 mix of EcCxGM:FGM:VascuLife medium and incubated for another 7 days on an orbital shaker (Infors HT, Celltron) at 50 rpm. PMNs were isolated from peripheral blood as previously described by Rajeeve et al. [24] from healthy donors after informed consent. For microscopy, the PMNs were stained by incubating with 0.5 μg/ml Carboxy SNARF-1 (ThermoFisher, S22801) for 20 min at 37°C protected from light. The models were flipped (basal side up) and 2.5x10^6^ PMNs in RPMI were pipetted to the base of 7 mm models. The PMNs were allowed to adhere for 1 h before the model was reflipped into the apical up orientation. To stimulate PMN migration either 100 ng/ml IL-6 (Sigma Aldrich, GF338) or infected epithelial cells were used as chemotactic signals.

### 2.8 Quantification of surface cells for infection

The number of cells of the most apical layer of the multilayered hEcCxM model was estimated from scanning electron micrographs using FIJI [42]. The average size of the cells was measured and extrapolated to the model surface area to determine the number of surface cells. This number was used to estimate multiplicity of infection (sc_MOI) for both pathogens (Figure S 4).

### 2.9 Bacteria culture and infection of mucosa models

*Neisseria gonorrhoeae* strain MS11 (ATCC, BAA-1833) was grown on *GC*-agar (ThermoFisher, CM0367) supplementedwith 1% vitamin mix [44] for 14-16 h. For infection, the bacteria were swabbed from the agar plate and resuspended in 10 ml advanced DMEM/F12 medium (ThermoFisher, 12634028) supplemented with 1% 100x Glutamax (ThermoFisher, 35050061) and 1% 1 M HEPES (ThermoFisher, 15630080) (ADF++) and their density determined by measuring OD_550_. Additionally, serial dilutions of OD_550_ 0.1 *GC* suspensions were plated to determine the number of viable *GC* for estimating the multiplicity of infection. The hEcCxM surface was washed once with ADF++ before infecting apically with 50 μl of the *GC* suspension in ADF++ with the respective sc_MOI and incubated for 6 h. The surface of the models was washed at 6 h post infection (hpi) with fresh ADF++ to remove non-adherent bacteria. For infections lasting longer than 30 h, the medium in the basal chamber was changed every 24 h. All incubations were done in a humidified incubator at 37°C and 5% CO_2._

*Chlamydia trachomatis* serovar K/BOUR (ATCC, VR-348B) was propagated by infecting HeLa-229 cells (ATCC, CCL-2.1) for 76 h. The infected cells were lysed with glass beads (Roth, A557.1) and the elementary bodies (EBs) were separated from the reticulate bodies (RBs) by gradient centrifugation in 1x sucrose-phosphate-glutamate (SPG) buffer as previously described by Vollmuth et al. [45]. *C. trachomatis* (*Ctr)* quantification was done using the Attune NxT flow cytometer (ThermoFisher) according to Klasinc et al. [46] and infectivity assays in HeLa-229 cells to determine sc_MOI. The hEcCxM model surface was washed once with ADF++, infected apically with 50 μl C*. trachomatis* serovar K (*CtrK)* suspension in ADF++, incubated for 6 h and nonadherent bacteria were washed away with fresh ADF++. For infections lasting longer than 30 h, the medium in the basal chamber was changed every 24 h.

### 2.10 Quantification of infection

Model associated *N. gonorrhoeae* (*GC)* was determined using colony forming units (CFU) assay. The tissue models were washed with DPBS, detached from the cell crowns and transferred into 2 ml screw cap tubes (Sarstedt, 72.694.005) containing 500 μl ADF++ and autoclaved glass beads. The tissue models were lysed by agitating with a Savant Bio 101 FastPrep FP120 homogenizer at 4 m/s for 30 s. 10-fold serial dilutions were made in sterile 1x Dulbecco's PBS (DPBS). 50 μl were then plated on *GC*-agar plates and cultured for 24 h in a humidified incubator at 37°C and 5% CO_2_. The number of colonies on the *GC*-agar plates were counted and expressed as colonies per model.

*Ctr* load was assessed by using quantitative PCR (qPCR) of the *omcB* gene. To isolate the genomic DNA, the tissue models were washed 3x with DPBS and transferred from the cell crowns into 1.5 ml safe seal tubes containing 100 μl QuickExtract DNA extraction solution (Biozym, 101098). Genomic DNA was extracted from the tissue models by incubating at 65°C for 6 min followed by 6 min at 98°C. The tube was then vortexed for 1 min and directly placed on ice. The tubes were centrifuged at 500 g for 1 min to remove cellular debris. The supernatant was transferred into clean 1.5 ml safe seal tubes and stored at −20°C until qPCR analysis. The qPCR was performed using the primers omcB:fwd 5′ GACACCAAAGCGAAAGACAACAC ′3 and omcB:rev 5′ CTCATGAACCGGAGCAACCT ′3). 2 μl of the tissue lysate was used in a 20 μl reaction mixture. The qPCR was performed according to the StepOne software (v2.3) using a GreenMasterMix (2x) High ROX (Gennaxon, M3052.0500) and the StepOnePlus system (Life Technologies). Quantification was done according to Lee et al., [47] using a pUC19 plasmid (Takara Bio, 638947) containing *omcB* and expressed as *Ctr* particles per model.

### 2.11 Infectivity assay for *Chlamydia trachomatis*

To quantify *Ctr* infectivity, the infected models were washed with DPBS, detached from the insert and transferred into 2 ml screw cap tubes containing 500 μl ADF++ and autoclaved glass beads. The tissue models were then lysed by agitating the models with glass beads using Savant Bio 101 FastPrep FP120 homogenizer at 4 m/s for 30 s 200 μl of the model lysates were mixed with 40 μl 5x SPG and stored at −80°C until reinfection. For reinfection HeLa-229 cells were seeded in 48 well plates in FGM. 24 h later the cells were treated with 1 μg/ml Cycloheximide (Sigma Aldrich, C4859-1 ML) and infected with 10-fold serial dilutions of the model lysates in triplicates and incubated for 48 h. The cells were fixed with 4% PFA and 5 random phase contrast pictures per well were taken using a Leica DMI 3000 B microscope (Leica). *Ctr* inclusions were counted using FIJI [42] and infectivity was expressed as infectious particles per model.

### 2.12 Cytokine analysis with cytometric bead array

Secreted epikines in the basal compartment were quantified using the LEGENDplex Human Inflammation Panel 1 (BioLegend, 74809). The supernatants were collected post infection, centrifuged at 2500 g for 10 min to remove cellular debris and stored at −80°C until measurement. The assay was performed as recommended by the manufacturer but with 10 μl volumes. The beads were captured on an Attune NxT (ThermoFisher) flow cytometer and cytokines were quantified using the online LEGENDplex Data analysis software suite.

### 2.13 Dissociation of cells and sequencing

To dissociate the cells from the hEcCxM models for single-cell RNA sequencing (scRNA-seq), the apical and basal compartments were washed with 100 μL and 2 mL PBS respectively. The models were then disassembled and transferred into 1 mL Accumax solution (Sigma Aldrich, A7089) in Eppendorf tubes. After a 10-min incubation at room temperature to allow tissue penetration, samples were incubated at 37°C for 1 h on a shaker (180 rpm), followed by brief vortexing at medium speed. Dissociated cells were then resuspended by pipetting and transferred to a 15 mL Falcon tube containing 4 mL of sterile wash buffer (PBS with 0.5% BSA). Cells were pelleted by centrifugation at 350*g* for 7 min. The supernatant was discarded, and the pellet was resuspended in 500 μL wash buffer for cell counting. For each donor, equal numbers of cells from two models were pooled to obtain sufficient material for sequencing. Cells were then resuspended in 5 mL sterile PBS and directly used for library preparation.

The cells dissociated from hEcCxM from three donors were combined and processed for single-cell RNA sequencing. The cells were pelleted by centrifuging (7 min, 1000 g, 4°C) and resuspended in PBS containing 0.04% w/v BSA (400 μg/ml) at a concentration of 1000 cells per μl. A 10x Chromium Controller was used to encapsulate single cells into nanolitre-scale Gel-Bead-In-EMulsions (GEMs) and Single-Cell 3′ Reagent Kit v3 for reverse transcription, cDNA amplification and library construction as specified by manufacturer. Approximately 13,200 cells per sample were loaded onto the controller and amplified using a SimpliAmp Thermal Cycler (Applied Biosystems). Libraries were then quantified with a QubitTM 3.0 Fluorometer (ThermoFisher), and quality was checked using a 2100 Bioanalyzer with a High Sensitivity DNA-kit (Agilent). Sequencing was performed in paired-end mode with S1 100-cycles kit using Novaseq 6000 sequencer (Illumina). Approximately 400 million reads were allocated per sample with at least 70,000 reads per cell.

### 2.14 Single cell RNA-Seq data analysis

Raw sequencing data were demultiplexed, aligned to the GRCh38 human genome assembly (Ensembl98), and unique molecular identifiers (UMIs) were quantified using CellRanger (v8.0.1; 10x Genomics). Cells were assigned to a donor using the souporcell algorithm (https://doi.org/10.1038/s41592-020-0820-1) based on the binary alignment map (BAM) file and barcodes exported by CellRanger. The count matrix was loaded into R (v4.2.3), and Seurat (v5.0.1) was used for downstream analysis. Only cells flagged as singlets by souporcell were retained for analysis. Further, low quality cells and potential doublets were filtered based on mitochondrial UMI count fraction per cell (<15%), UMI counts per cell (>2000, <40000), as well as detected genes per cell (>1000, <6500). Counts were log-normalized (NormalizeData, default parameters), and highly variable genes (HVGs) were selected (FindVariableFeatures, default parameters). HVGs were scaled (ScaleData, default parameters) and principal component analysis (PCA) was computed (RunPCA, default parameters). The Seurat reciprocal principal component analysis (RPCA) workflow with the donors as covariate was applied for batch correction. Based on the RPCA, a nearest neighbor graph was constructed (FindNeighbors, dims = 1:30), and the uniform manifold approximation and projection (UMAP) algorithm was used to compute a two-dimensional embedding (RunUMAP, dims = 1:30). Unsupervised clustering was performed using the Louvain algorithm (FindClusters, resolution = 0.7)**.** Differentially expressed genes between clusters were identified using the Wilcoxon rank-sum test (FindAllMarkers, min.pct = 0.2), and clusters were annotated according to known marker genes and their gene expression profiles.

### 2.15 Trajectory and pseudotime analysis

Developmental trajectories of the epithelial compartment of the hEcCxM model was reconstructed from the hEcCxM models using URD(v.1.1.1) [48]. Destiny (v.3.9.1) [49,50]was used to compute the diffusion map using default parameters. Pseudotime was calculated using the built-in functions from a defined root population corresponding to the basal Epi KRT5+ cells. Potential trajectory endpoints (Tip) for terminally differentiated epithelial cell subtypes were characterized by the expression of suprabasal and superficial markers (*KRT13, IVL*). The URD tree topology was constructed with “buildTree” with parameters to minimize spurious branching. Temporal gene expression dynamics along each trajectory was modeled using “plotSmoothFit” function.

### 2.16 Integrative analysis with native cervical tissue scRNA-seq data

For integrative analysis, single-cell transcriptomic datasets from the hEcCxM models were compared to 5 publicly available healthy ex-vivo cervical tissue datasets from three studies: E-MTAB-11948 (ArrayExpress, 2 samples) GSE168652 (GEO,1 sample) and GSE208653(GEO, 2 samples) [[51], [52], [53]]. Each dataset was preprocessed independently in R using Seurat v5.5.0 [54]. The adaptive median absolute deviation (MAD)-based thresholds quality control method was used to retain cells with feature counts between 200 and sample-specific upper limit (media ± 3xMAD log-transformed feature counts) and mitochondrial reads above 20%. Each dataset was independently normalized using SCTransform (v2, glmGamPoi), regressing the mitochondrial fraction, and selecting the 3000 most highly variable genes. The datasets from the hEcCxM models (7822 cells) and ex-vivo cervical tissues (46114 cells) were then merged into a single Seurat Object using the “merge” function and principal component analysis (PCA) was performed on the shared highly variable genes across 50 principal components. Batch correction was performed using Harmony (v2.0.3). A shared nearest-neighbor graph (FindNeighbors) was constructed based on the top 30 Harmony-corrected principal components and a Louvain community detection with a resolution of 0.5 was applied using the “FindClusters” function. Visualization of the two dimensional reduction was performed with “RunUMAP” function. Cluster identities were assigned using a score-based approach. Module scores were computed for curated canonical marker genes using the “AddModuleScore” function. Each cluster was assigned the identity corresponding to its highest mean score across according to major cervical cell types and validated against Human Protein Atlas database [55]. The squamous epithelia (*TP63, KRT5, KRT13*) and fibroblast lineages (*DCN, LUM, COL1A1*) were subsetted, since the ex-vivo tissue dataset contained other cell types such as immune, endothelial and smooth muscle cells (Figure S 6C) for downstream analysis. Each lineage subset was independently re-clustered following the same PCA-Harmony-UMAP integration workflow. Count layers were consolidated using “JoinLayers” function. To assess bulk transcriptional fidelity of the hEcCxM relative to native ectocervical tissue, pseudobulk expression profiles were constructed per lineage. Pairwise Spearman rank correlation coefficients were computed between all pseudobulk profiles. Compositional differences between the hEcCxM model and native EcCx was computed using Jensen-Shannon divergence , calculated on a log base 2 scale bounded between 0(identical distribution) and 1(maximally dissimilar).

### 2.17 Statistical analysis

The data were organized in Microsoft 365 Excel. Statistical analysis and graphs were generated with GraphPad Prism v10 for windows (GraphPad Software). P-values were determined using one-way or two-way analysis of variance (ANOVA) followed respectively by post-hoc Tukey and Sidak where appropriate.

## 3 Results

### 3.1 The organotypic ectocervical mucosal models show typical characteristics of native human ectocervix mucosa

We present a co-culture human ectocervix mucosal (hEcCxM) model derived from patient-matched stromal and epithelial cells seeded on a plastically compressed collagen gel as described by Reuter et al. for a human skin model [40,41]. Plastically compressing the collagen gel to high-density prevented its contraction, thereby preserving tissue structure as described by Reuter et al., [40,41]. As shown in Fig. 1B, fibroblasts were embedded in the scaffold to improve mucosa differentiation [35,[56], [57], [58]]. A custom-designed cell crown (Figure S 3A**)** enabled the static culture of the hEcCxM models in 12 (Figure S 3D**)** and 24-well formats. The co-culture models were differentiated at an air-liquid-interface and assessed histologically at 21 days (Fig. 1B). Haematoxylin and Eosin (H&E) (Fig. 1C1) staining of formalin fixed paraffin embedded (FFPE) sections showed a stratified squamous epithelium with a basal-apical polarization. Periodic-Acidic-Schiff stain (PAS**)** (Fig. 1C2) further confirmed the accumulation of glycogen, a key structural feature of the *ex vivo* tissues [59]. Transmission electron microscopy (TEM) showed an organized epithelial architecture, including a flattened top layer, an intermediate layer of rounded cells and a basal layer of elongated cells with interdigitating finger-like projections (Fig. 1D). Immunofluorescence staining of the paraffin sections showed typical honeycomb organization of the junctional protein E-cadherin. Furthermore, the expression pattern of EcCx markers such as *TP63*, cytokeratin 5 (*KRT5*) and cytokeratin 13 (*KRT13*) recapitulated *ex vivo* tissue (Fig. 1C). The presence of fibroblast in the plastic collagen gel was confirmed with vimentin (VIM) staining.

To further characterize the hEcCxM models, scRNA-seq analysis was performed on models derived from 3 different donors. Uniform Manifold Approximation and Projection (UMAP) analysis with canonical markers identified 8 distinct cell subtypes which clustered broadly into epithelial and fibroblast lineages (Fig. 1E). The different cellular subtypes were present and clustered together from the hEcCxM models developed from three different donors (Fig. 1F, Figure S 5), demonstrating consistent tissue composition across individuals. Using canonical cell type markers, the epithelial lineage subclustered further into basal (*CAV1, KRT14, KRT5, TP63*), intermediate (*KRT13, SERPINB3*) and upper layers (*IVL, KRT4, MUC1, PI3*). The fibroblast lineage (*COL1A1, COL6A3*) lineage was divided into the proliferative fibroblasts (*MKI67, CAVIN1*) and myofibroblasts (*SPON2, POSTN, ACTA2*) populations. Furthermore, we observed markers such as *KRT6A*, *KRT17* and *ESR1* which, despite being present in the epithelial tissue, showed decreasing expression with increasing differentiation from the basal layer (Fig. 1G).

Pseudotime analysis of the hEcCxM models showed two developmental trajectories of the epithelial population originating from the basal cell cluster (Epi_KRT5+)(Fig. 1H). The first trajectory projected towards the proliferating cluster (Epi KRT5+ Prolif), consistent with progenitor self-renewal and basal layer maintenance. The second progressed toward terminal differentiation (Epi_2) through transcriptionally intermediate population, recapitulating the stepwise stratification program characteristic of the native ectocervical epithelium. The expression of basal progenitor markers *KRT5* and *TP63* decreased with increasing pseudotime, while suprabasal and superficial differentiation markers *KRT13*, *MUC1* and *IVL* were progressively upregulated, marking the transition from active cell cycling toward terminal differentiation. The inverse relationship between the basal and suprabasal gene expression along the pseudotime axis mirrors the spatial stratification organization typical of the native ectocervical tissue.

To assess the degree to which the hEcCxM model recapitulates the *in vivo* transcriptional profile of the native ectocervix, data from the hEcCxM models was integrated with 3 publicly available human cervical tissue scRNA-seq datasets [[51], [52], [53]]. Unsupervised clustering following Harmony-based batch correction revealed that clustering of the epithelial and fibroblast populations were driven by transcriptional identity rather than origin of the dataset (Fig. 1I, Figure S6 A, B), thus confirming biological coherence of shared cell type. The squamous epithelia and fibroblast populations were subsequently subsetted from the integrated object to further characterize the transcriptional similarity of the native ectocervical tissue and hEcCxM models (Fig. 1J). UMAP of the epithelial subset resolved five epithelial subpopulations: basal, proliferating, parabasal, suprabasal and superficial-annotated to reflect stratification hierarchy and to distinguish them from the unintegrated hEcCxM-only analysis. The fibroblast lineage clustered into fibroblasts, myofibroblasts and proliferating fibroblasts (Fig. 1K). Examination of the per-cluster cell proportions confirmed that the hEcCxM contributed to all the identified epithelial and fibroblast subtypes (Fig. 1J). Pseudobulk Spearman rank correlation analysis between native tissues and hEcCxM subtypes demonstrated strong transcriptional concordance with a correlation coefficient of approximately 0.70 across epithelial and fibroblast lineages (Fig. 1M). Taken together, our histological, immunostaining and integrative scRNA-seq data demonstrate that the hEcCxM model closely recapitulates the transcriptional and cellular architecture of the native human ectocervical mucosa.

### 3.2 *Neisseria gonorrhoeae* adheres to and invades hEcCxM models

**Fig. 2:**
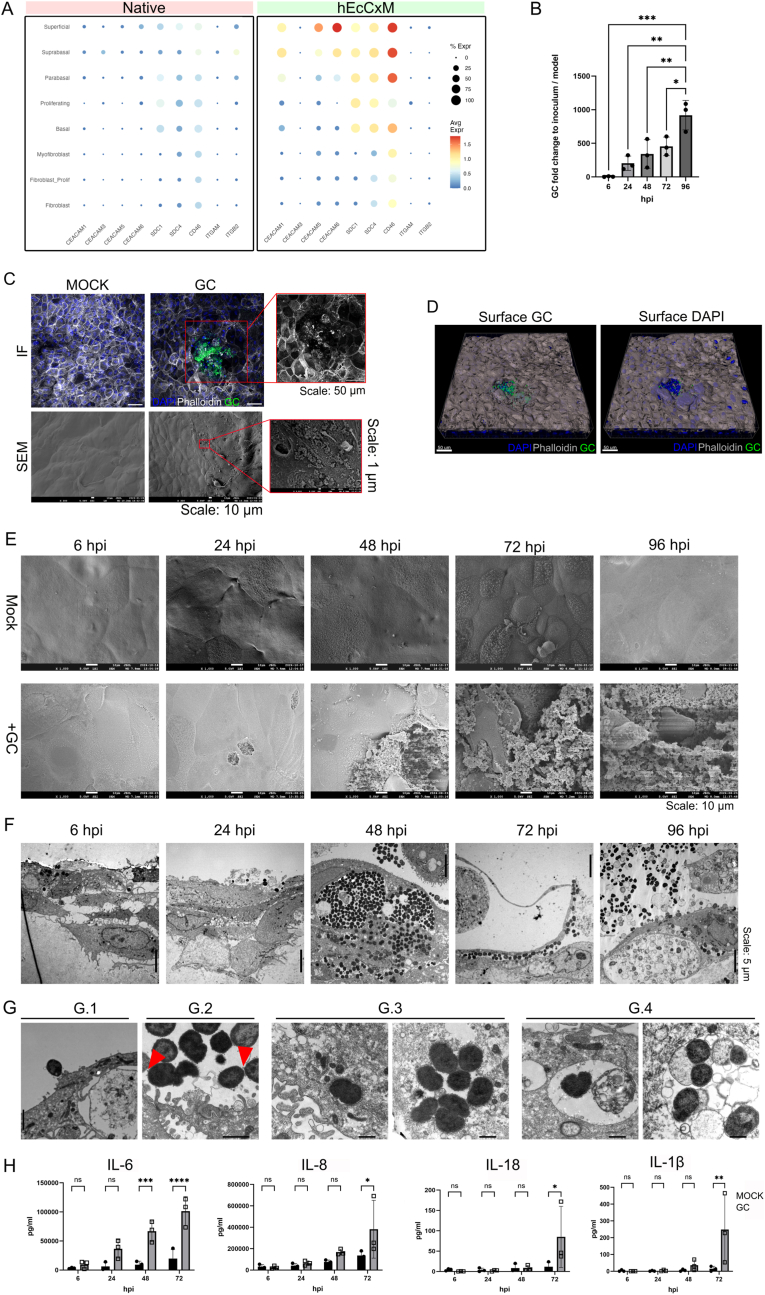
***Neisseria gonorrhoeae* colonizes the hEcCxM model. A:** Dot plot of the expression of markers of interest for *GC* infection non-infected hEcCxM models and native tissues after integration. Dot size represents the proportion of non-zero expressing cells in each group. **B:** Temporal *GC* fold change relative to inoculum from CFU assays for 96 h. Data represents mean ± sd of triplicates of 3 independent experiments from 3 donors (N = 3). A one-way ANOVA with Tukey's multiple comparisons test was performed (∗p ≤ 0.05, ∗∗p ≤ 0.01, ∗∗∗p ≤ 0.005). **C:** Confocal laser scanning micrographs and scanning electron micrographs (SEM) of 30 h *GC* infected hEcCxM model showing bacterial microcolonies and loss of epithelial integrity. **D:** Surface rendering of *GC* microcolonies associated with a strong DAPI signal indicating potential biofilm formation. **E:** Scanning electron micrographs showing *GC* colonization and tissue damage (bottom panel) compared to mock treated (top panel) tissues at different timepoints. **F:** Transmission electron micrographs showing *GC* in the intra and paracellular space of epithelia at different timepoints post infection. **G:** Transmission electron micrographs of different *GC* infection phenotypes: G.1: *GC* associated with a surface cell showing a bulge towards the *GC* (Scale: 2 μm) G.2: hEcCxM infection with *GC* associated with small vesicle structures (red arrowhead) (Scale: 1 μm) G.3: Intracellular *GC* within membranes (Scale: 500 nm) G.4: Intracellular *GC* within membranes with fused vesicles (Scale 500 nm) **H:** Cumulative secreted proinflammatory cytokines in basal medium at different timepoints of *GC* infection. Data represents mean ± sd of 3 independent experiments from 3 different donors. A two-way ANOVA with Šídák's multiple comparisons test was performed (∗p ≤ 0.05, ∗∗p ≤ 0.01, ∗∗∗p ≤ 0.005, ∗∗∗∗≤0.001, ns nonsignificant). (For interpretation of the references to color in this figure legend, the reader is referred to the Web version of this article.)

To investigate the susceptibility of the hEcCxM to *GC* infections, we examined the expression of key host receptors that mediate *GC* colonization across the integrated dataset, including carcinoembryonic antigen-related cell adhesion molecules (CEACAM) and surface proteoglycans [30,[60], [61], [62]]. We observed that *CEACAM1*, *CEACAM5* and *CEACAM6,* targets of the *GC* opacity (Opa) proteins*,* were mainly expressed in the parabasal, suprabasal and superficial layer of the hEcCxM models compared to the native tissue, suggesting an enhanced receptor availability in the model epithelium. The heparan sulfate proteoglycans, *SDC1* and *SDC4*, which mediate adherence and invasion via Opa, were broadly expressed in all epithelial cell subpopulations of both native and hEcCxM models. However, their average expression was higher in the hEcCxM models compared to the native ectocervix tissues after integration. *CD46*, implicated in type IV pilus binding [63,64], was expressed in all the epithelial cell clusters in both con (Fig. 2A). In contrast, *ITGAM* and *ITGB2*, the α and β subunits of the complement receptor 3 (CR3), were absent from the hEcCxM while native tissues showed low-level expression (approximately 0.5-fold) in the suprabasal epithelial cells (Fig. 2A). This expression is consistent with the established predominant expression by innate immune cells [55]. Collectively, the expression of *GC*-relevant receptors across both the hEcCxM models and native tissues are broadly harmonious, thus supporting the utility of the model as a representative platform for investigating gonococcal-host interactions.

To validate the application of the hEcCxM model for gonococcal infections implied by the receptor expression profile described above, the models were apically infected with *GC* at a surface cell-based multiplicity of infection (sc_MOI) of 0.15 and host-pathogen interactions were assessed at 30 h post infection (hpi) by confocal laser scanning microscopy (CLSM). Consistent with the broad expression pattern of receptors, *GC* successfully colonized the apical hEcCxM model and formed microcolonies by 30 hpi (Fig. 2C, upper panel). Interestingly, despite a 6 h incubation with the *GC* suspension prior to re-establishing the air-liquid interface, the infections were restricted to regions of the tissue with disrupted phalloidin staining (Fig. 2C, upper panel). This observation was confirmed by SEM analysis, which showed *GC* microcolonies confined to damaged epithelia areas while regions lacking colonizing bacteria appeared intact (Fig. 2C, lower panel). 3D surface reconstruction of the infected hEcCxM models demonstrated spreading of the *GC* towards the basal layer and underneath neighboring epithelial cells. Additionally, *GC* accumulation within the tissue was associated with a strong DAPI signal (Fig. 2D) indicative of biofilm formation.

To assess the potential use of the hEcCxM model for long term *GC* infections, tissues were infected at a sc_MOI of 1 and bacterial burden was monitored daily for 96 h. CFU assay was performed every 24 h with initial adherence assessed at 6 hpi. A time-dependent increase of *GC* infection of up to 918-fold at 96 hpi was observed (Fig. 2B). Lesions appeared as early as 24 hpi and expanded in a time-dependent manner over the 96 h, correlating with bacterial load and tissue damage (Fig. 2E, Figure S 1**).**

TEM showed *GC* inside the host cells and in paracellular spaces as early as 6 hpi. At 48 hpi large accumulations of *GC* were present in membrane bound compartments, while some less electron dense *GC* were also observed in the cytoplasm. At 72 hpi, *GC* were predominantly found in the paracellular space while at 96 hpi, major tissue damage was observed with *GC* detected throughout the epithelial layer (Fig. 2F). Distinct infection phenotypes described for natural *GC* infections of the cervix [17] were observed (Fig. 2G) including bulging of host cell membrane towards adherent *GC* (**G.1**), *GC* associated with vesicular structures (**G.2**, red arrowhead), intracellular membrane-bound *GC* (**G.3**) and *GC* clusters associated with vesicular structures (**G.4**).

*GC* infection of the cervix induces the secretion of inflammatory cytokines. To investigate this response in the tissue models, supernatants from the basal compartment of infected hEcCxM was collected and analyzed for secreted cytokines using the Biolegend Legendplex multiplex immunoassay kit. For infections longer than 24 h, cytokines were cumulatively measured to assess the total cytokine production over time. Cytokine concentrations were compared to mock infected models for each timepoint. A significant increase in IL-6 secretion was observed at 48 hpi and 72 hpi in infected hEcCxM models. Additionally, IL-8, IL-1β and IL-18 were significantly elevated at 72 hpi (2-way-Anova). In contrast, levels of IL10, IL-17A and MCP-1 showed no significant change compared to control (Figure S 2A). In summary, the hEcCxM model robustly recapitulates key aspects of the *GC*-host interactions, making it a valuable tool to assess *GC* pathogenicity and host response.

### 3.3 *Chlamydia trachomatis* infects and replicates in hEcCxM models

**Fig. 3:**
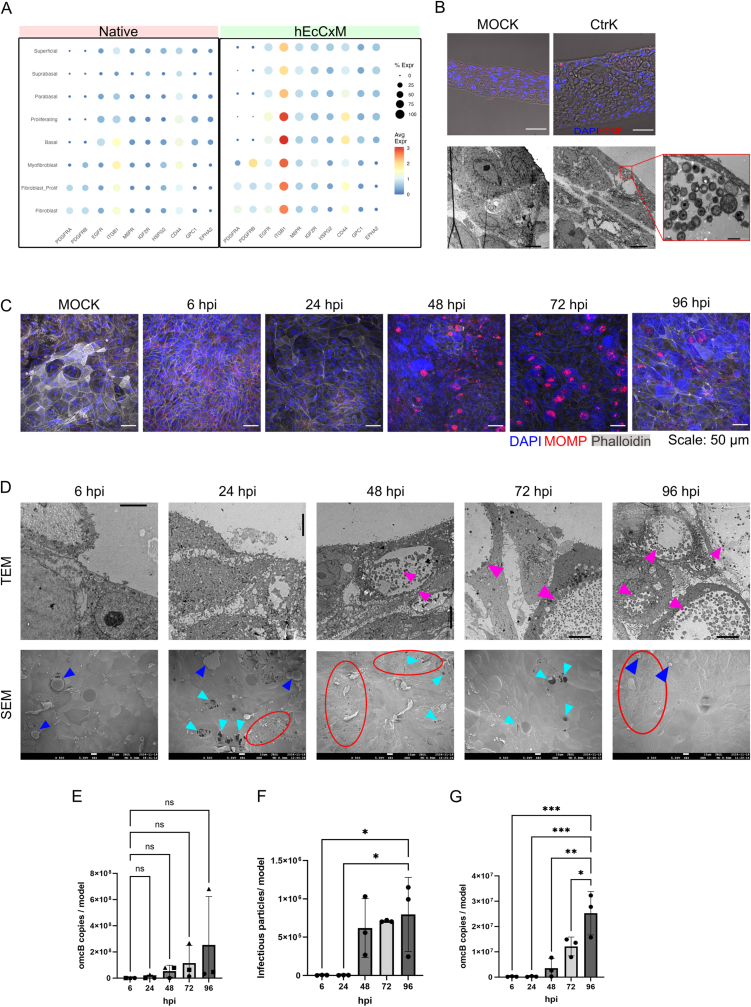
***Chlamydia trachomatis* infects apical cells of the hEcCxM model and completes its developmental cycle: A:** Dot plot showing the expression and distribution of markers of interest for *Ctr* infection in hEcCxM and native tissue after integration. Dot size represents the proportion of non-zero expressing cells in each group. **B:** CLSM and TEM images of 30 h *Ctr* infected hEcCxM models showing inclusions in apical cells (Scale bar: 50 μm and 10 μm respectively). **C:** Representative images of maximum projection of wholemount immunofluorescent images of *Ctr* (MOMP) infected and MOCK hEcCxM showing *Ctr* inclusions at different timepoints post infection (Scale bar: 50 μm). **D:** Representative transmission electron micrographs (upper panel) show no observable inclusions at 6 and 24 hpi while heterogenous inclusions containing EBs and RBs (magenta arrowheads) were observed at 48, 72 and 96 hpi. Representative scanning electron micrographs (bottom panel) showing shed cells (blue arrowhead) and lesions (cyan arrowhead) and partially ruffled surface (red circle) at different timepoints post Ctr infection hpi. At 72 hpi, model surface seems to indicate less surface ruffling although some lesions were present (Scale bar: 10 μm). **E:***CtrK* genome copies per model over 96h using *omcB* as reference. Data represents mean ± sd of 3 independent experiments done in duplicates from 3 different Donors. A one-way ANOVA with Tukey's multiple comparisons test was performed (ns = non-significant). **F:** Infectivity assay of *CtrK* from lysed infected hEcCxM show donor-dependent effect on *Ctr* particles. Data represents mean ± sd of 3 independent experiments done in duplicates from 3 different donors. A one-way ANOVA with Tukey's multiple comparisons test was performed (∗, p < 0.05). **G:***CtrK* genome copies per model from hEcCxM models developed from a single donor at different timepoints using *omcB* as reference. Data represents mean ± sd of 3 independent experiments done in duplicates from one Donor. A one-way ANOVA with Tukey's multiple comparisons test was performed (∗p ≤ 0.05, ∗∗p ≤ 0.01, ∗∗∗p ≤ 0.005, ns = non-significant). (For interpretation of the references to color in this figure legend, the reader is referred to the Web version of this article.)

*Ctr* adhesins interact with several host receptors, including surface heparan sulfate glycosaminoglycan (HSPG) and estrogen receptor complexes in order to facilitate entry into non-phagocytic cells [65,66]. We therefore evaluated the expression of a set receptors that mediate *Ctr*-host interaction in the integrated dataset (Fig. 3A). *EGFR* [67,68] and *ITGB1* [69] were most highly expressed in the basal epithelial subpopulation but were also present across all epithelial and fibroblast lineages. *ITGB1* expression was notably highly expressed in the hEcCxM models (∼2.5-fold) compared to the native tissues (∼1.5-fold), with the highest average expression observed in basal cells and fibroblasts in both tissue types. The Ephrin type-A receptor 2 (*EPHA2)* which mediates *Ctr* adhesion and subsequent internalization [70], was exclusively expressed in the epithelial lineage with a higher proportion of expressing cells in the hEcCxM models compared to native tissue (Fig. 3A). *HSPG2* and *GPC1*, two HSPGs [[71], [72], [73], [74], [75]], were similarly restricted to the epithelial lineage in both conditions. *CD44*, a receptor modified by the *Ctr* type III secretion system [76] to promote invasion, was broadly expressed in both the epithelial and fibroblast lineages.

In both tissues, *PDGFRA* and *PDGFRB* [77] were not expressed in the epithelia but in the fibroblast lineage, which is consistent with their established stromal expression. *M6PR* and *IGF2R*, two mannose-6-phosphate receptors whose interaction with nexins is disrupted by Chlamydia to facilitate intracellular entry [78], were detected across both epithelial and fibroblast lineages (Fig. 3A). Overall, the markers show similar pattern of expression in cellular subtypes despite differences in their average expression and the proportion of expressing cells between native and hEcCxM models.

To validate the application of the hEcCxM models as a platform for *Ctr* infections, they were infected with *Ctr* serovar K (*CtrK*) at a sc_MOI of 40 and assessed at 30 hpi. CLSM and TEM analysis revealed that *Ctr* inclusions were restricted to the uppermost epithelial layer. TEM further identified both small electron dense EBs and larger less dense RB within the *Ctr* inclusions (Fig. 3B) indicating completion of the *Ctr* life cycle within the hEcCxM models. To assess the host response and applicability of the hEcCxM models for long term *Ctr* infections, we infected with *CtrK* and monitored the tissues daily for 96 h. No discernible inclusions at 6 and 24 h were observed. From 48 hpi to 96 hpi, inclusions became visible with an uneven distribution and size (Fig. 3C, D, Figure S 1B). TEM images indicated asynchronous development with smaller inclusions mainly containing RBs (magenta arrowhead) while larger inclusions contained a mixture of RBs and EBs (Fig. 3D). Quantitative PCR analysis targeting *omcB* demonstrated a time-dependent increase in *Ctr* copies. However, this increase was not statistically significant when comparing the different timepoints and models derived from different donors (Fig. 3E). No discernible inclusions were observed from infectivity assays with lysates from tissues infected for 6 and 24 h, while inclusions were observed when samples were collected from tissues infected for 48 to 96 h. The *CtrK* from 96 hpi lysates showed significantly higher infectivity (p = 0.035) compared to lysates from 6 to 24 hpi. Lysates from 72 hpi showed a trend towards significance (p = 0.064) compared to early timepoints (Fig. 3F). To determine if the high variability in the *omcB* genome copy assay were due to donor variations, we repeated the experiment with models developed from a single donor. The *omcB* genome copy from 96 hpi infected tissues was significantly higher (p ≤ 0.05) compared to all the other timepoints (Fig. 3G) indicating the influence of donor on infectivity. SEM images showed shedding of surface cells already at 6 hpi, while lesions were first seen on the tissue surface at 24 hpi (cyan arrowhead). Interestingly, these lesions appeared to be closed at later time points of the infection (Fig. 3D).

To investigate the inflammatory response of the hEcCxM models to *Ctr* infections, the media from the basal compartment was collected and analyzed with BioLegend legendplex multiplex immunoassay kit. The supernatants were assessed for IL6, IL8, IL17-A, CCL2(MCP1), TNFa, IL18 and IL10, with cytokine concentrations expressed cumulatively due to daily media change. *Ctr* infection did not seem to induce significant secretion of the assessed cytokines into the basal medium when compared to the mock infected controls at the individual time points (Figure S 2B). Taken together, the data demonstrate that the hEcCxM model expresses a repertoire of receptors essential for *Ctr* infection, supports the complete chlamydial developmental cycle and sustains productive infection over an extended time course*.* The model has therefore demonstrated its utility for investigating *Ctr* pathogenesis and host-pathogen dynamics in a physiologically relevant epithelial context.

### 3.4 Integration of neutrophils into the hEcCxM

In addition to their role as a physical barrier against microorganisms, the epithelial layer contributes to innate immunity by secreting anti-microbial peptides, expressing pattern recognition receptors (PRR) such as the toll-like receptors (TLR) and secreting cytokines and chemokines [79,80]. TLR on epithelial cells detect microbial components or tissue damage and induce the expression of cytokines and chemokines to recruit immune cells such as neutrophils and dendritic cells into the mucosa (reviewed in Ref. [81]).

**Fig. 4:**
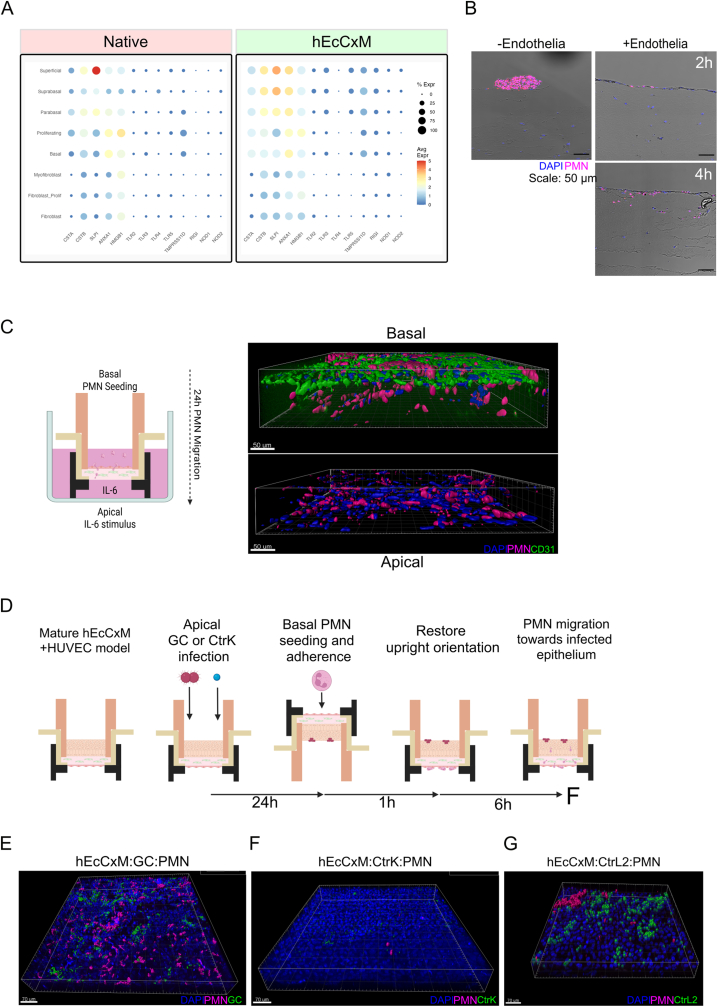
**Integration of primary PMN into the hEcCxM. A:** Dot plot of the expression of secretory genes and PRRs essential for epithelial immunity in hEcCxM and native tissue after data integration. Dot size represents the proportion of non-zero expressing cells in each group. **B:** Overlay of bright field and immunohistology of 5 μm sections of formalin fixed paraffin embedded hEcCxM models showing PMN interaction with collagen scaffold without and with an endothelial (HUVEC) cell layer. **C:** Schematic (created with www.biorender.com) and CLSM rendering of proof of principle experiment to assess PMN (red) migration through plastically compressed collagen scaffold with an endothelial layer (green). **D:** Schematic showing infection and addition of PMN to the hEcCxM models to assess PMN transmigration to sites of infection (created with www.biorender.com). **E:** Surface rendering of CLSM infected hEcCxM models showing the presence of PMN(red) in *GC*(green) after 30 hpi **F**: Surface rendering of CLSM of infected hEcCxM models showing delayed inclusion formation and PMN(red) recruitment to epithelial surface in hEcCxM models infected with *Ctr* serovar *K(*green). **G.** Surface rendering *of Ctr* serovar L2 showing the presence of inclusions (green) with PMNs (red) into epithelia after 30 hpi. (For interpretation of the references to color in this figure legend, the reader is referred to the Web version of this article.)

We examined the integrated dataset for expression of some *PRR*s as well as markers of anti-microbial peptides. As shown in Fig. 4A, tissue types showed similar expression patterns in both the fibroblast and epithelial lineages**.** Relatively few cells were observed from both native tissues and the hEcCxM models to express *TLR, NLR* and *RIG* (Fig. 4A) with less than 1-fold expression of *TLR3*, *TLR5* and *RIG1,* suggesting limited but detectable innate immune surveillance capacity of the ectocervix epithelium (Fig. 4A). A large proportion of the cells in both tissues expressed anti-microbial peptides, including *CSTA*, *CSTB*, *SLPI*, *ANXA1* and *HMGB1* (Fig. 4A). With the exception of *HMGB1,* the highest expression of the antimicrobials was observed in the suprabasal and superficial cell clusters. *TMPRSS11D* expression was restricted to the epithelial lineage with the highest expression in the intermediate epithelial population (Fig. 4A).

In addition to the epithelial cells, circulating professional phagocytic cells such as macrophages, dendritic cells and neutrophils can be found in the cervical mucosa (reviewed in Ref. [82]). To render the hEcCxM models immunocompetent, we added peripheral blood-derived polymorphonuclear neutrophils (PMN) to the basal compartment. To determine if the PMN could migrate through the compressed collagen scaffold, PMNs were stained with SNARF and seeded apically on the scaffold, without an epithelial and endothelial layer. Exogenous IL6 was added to the bottom compartment as a chemotactic stimulus. In the absence of endothelial cells, we observed that the PMNs aggregated at the surface and failed to migrate into the scaffold when directly seeded onto the collagen scaffold (Fig. 4B). However, when the scaffold was covered with a layer of endothelial cells (HUVECs), the PMNs adhered and successfully migrated into the scaffold after 4 h incubation (Fig. 4B). PMNs were detected at the bottom of the scaffold and in the underlying well-plates (data not shown) after 24 h (Fig. 4C) confirming their ability to transmigrate through the 1 mm thick scaffold. We then infected the hEcCxM models apically with *Ctr* and *GC* for 24 h, added the PMNs basally and incubated for 1 h to promote PMN adherence. Induction of the PMN transmigration towards the sites of infection was assessed via confocal microscopy after 6 h (Fig. 4D). PMNs were observed in the epithelial layers and in association with *GC* (Fig. 4E) when added basally, indicating active migration towards the infection. In contrast, *CtrK*-infected models showed minimal PMN presence in the infected epithelium (Fig. 4F), suggesting a differential capacity to induce neutrophil recruitment. To determine if the observation was serovar-dependent, the hEcCxM models were infected with the *C. trachomatis* serovar L2 for 24 h and PMN migration assessed as for *CtrK*. As shown in Fig. 4G, *C* *trachomatis* serovar L2 inclusions appeared to be bigger compared to the *CtrK* with subsequent observed PMN migration to epithelial. These findings suggest that both the *Chlamydia* serovar and the inclusion size may influence the recruitment of PMNs to the epithelial site of infection.

## 4 Discussion and conclusion

The pathogenicity of *Ctr* and *GC* has been extensively studied using 2D cell culture models which lack the cellular heterogeneity, polarity, and microphysiology of native tissues. As a result, key aspects of host-pathogen interactions in the human host remain poorly understood. In this study, we present an organotypic 3D coculture model of the normal human EcCx mucosa which closely recapitulates the cellular heterogeneity, polarity and organization of native human ectocervical mucosa for infection research. The hEcCxM model displays a stratified organization and a connective tissue layer consisting of donor-matched fibroblasts with cellular subtypes, which is consistent with native ectocervix epithelium differentiation [83] but with reduced layer thickness and less defined laminar boundaries. Its transwell-like configuration provides direct access to the luminal surface enabling simulation of natural infection. The addition of fibroblasts to the collagen scaffold promoted epithelial proliferation and differentiation through the secretion of factors such as HGF [34,35,59,84]. De Gregorio et al. [58] successfully developed a scaffold-free full thickness ectocervix mucosal model which was supported by extracellular matrix secreted from cervical fibroblasts. A previous study by McKinnon et al. [59] also reported that murine embryonic feeder cells and ovaries were necessary for stratification and maturation of an engineered EcCx mucosa model. However, by using a medium optimized for cervix organoid formation [39], we successfully developed a fully differentiated hEcCxM model without the need of murine fibroblasts in our collagen scaffold. The maturation of the hEcCxM model was confirmed through immunostaining and scRNA-seq showing expression and localization of key differentiation markers *KRT5, TP63* and *KRT13* (Fig. 1C, Figure S 6E) as well as the decreasing intensity of the junctional protein E-cad (Fig. 1C), from basal to suprabasal layers [2].

We showed that our hEcCxM model faithfully recapitulated transcriptional features of native tissues by integration of the single-cell transcriptomics (Fig. 1J–M, Figure S 6). At the single cell identity level, the models demonstrate high fidelity to native ectocervix mucosa cell types, which was confirmed by a 96.3% agreement between two independent classifiers (Seurat and SingleR) (Table S 3). However, compared to native tissue, the hEcCxM model showed altered average expression and proportions of expressing cells. This may reflect both genuine biological differences between native tissues and the hEcCxM model as well as integration-related bias. Biologically, the elevated expression likely reflects the absence of competing stromal and immune cells in the hEcCxM model environment [85]. In-depth analysis of the fibroblast subcluster revealed other fibroblast subtypes such as stromal, perivascular and inflammatory populations in the native tissue while the hEcCxM models were dominated by the stromal subtype (data not shown). Integration-bias is due to correction of technical variation rather than preserving raw expression magnitudes from different datasets [86,87]. The divergence between our model and the native tissue was reflected in the moderate pseudobulk Spearman correlation, which is consistent with benchmarking studies of *in vitro* human models [88] which define the boundary for interpretation of such an integrative analysis. This is further confirmed by the Jensen-Shannon divergence score of 0.693 (Table S 3), indicating differences in the tissue cellular complexity.

The ectocervix epithelium is normally covered by cervicovaginal fluid, which contains mucus, epikines, immunoglobulins, and antimicrobial peptides that contribute to the mucosal barrier against pathogens [[89], [90], [91]]. One such antimicrobial peptide, *SLPI*, has been shown to be bactericidal against *GC* by directly binding to the opacity protein [92]. In addition to serving as a physical barrier, the EcCx mucosa also expresses pattern recognition receptors (PRR) that detect pathogen-associated molecular patterns and damage-associated molecular patterns (DAMPs) to initiate an inflammatory response and recruiting professional phagocytes such as macrophages or neutrophils. Our hEcCxM model expresses key PRRs including Toll-like receptors (TLR), NOD like receptor (NLR) and RIG like receptors (Fig. 4A). In the FRT, TLR expression varies across anatomical sites and fluctuates with hormonal status [[93], [94], [95]], reflecting a balance between protective immunity and limiting inflammation-induced tissue damage. TLR2 and TLR4 are more highly expressed in the upper FRT compared to the lower FRT likely due to the increased microbiome in the lower FRT [96]. In our non-infected hEcCxM models, we observed minimal mucous (MUC1) production (Fig. 1C3) and detectable expression of antimicrobial genes. However, expression of *TLR*, *NLR* and *RIG* was low in both the native tissue and the hEcCxM models suggesting that this may be an intrinsic feature of the ectocervix. This pattern may reflect a reliance on physical barrier defenses to prevent pathogen entry rather than on a sentinel-based immune surveillance approach [97].

Adherence and invasion of the epithelia by *GC* is mediated by the interaction of Opa and type IV pilus with CEACAMs [28,[98], [99], [100]] and heparan-sulfate proteoglycans expressed on the surface of the host cells [30,[101], [102], [103], [104]]. Additionally, *CD46*, which is ubiquitously expressed in cells has been implicated as a pilus receptor on epithelial cells [105,106]. Single cell RNAseq of our non-infected hEcCxM models confirmed high expression of *CEACAMs* 1,5,6 in the apical layers (Fig. 2A). In addition, we confirmed the expression of heparan-sulfate receptors *SDC1* and *SDC2* in basal, parabasal and apical layers [16,17,21]. Upon infection, *GC* formed microcolonies on the tissue surface and displayed several aspects of the natural infection such as outer membrane blebbing, vesicle formation, and membrane ruffling [16,17,19] *GC* was observed in the intra and paracellular space as early as 6 hpi with visible clusters at 48 hpi. Consistent with *in vivo* observations, *GC* formed biofilm-like structures associated with extracellular DNA and networks of bacterial membrane [[19], [20], [21]]. Biofilm-like matrices as well as a strong DAPI signal were observed in infected models as well.

*GC* infections are known to induce a systemic increase in cytokines without a corresponding rise in local protective immunity in the cervix [60,61]. *GC* infections of the cervix induce proinflammatory cytokine secretion and subsequent infiltration of neutrophils which are primarily responsible for the clinical symptoms [10,27,28]. Infection of the hEcCxM induced significant secretions of IL6 and IL8, IL18 and IL1β into the basal supernatants at different time points post infection. Additionally, *GC*-infected models induced the migration of neutrophils to the sites of infection, mimicking *in vivo* immune responses. We observed that prolonged infections with *GC* led to epithelia degradation as observed in prolonged natural infections.

Basal cells are more susceptible to *Ctr* infection compared to fully differentiated cells especially in stratified tissues such as the EcCx and vagina. *Ctr* adherence and invasion of epithelial cells is mediated by heparan sulfate and integrin ß receptors [65,107]. The reported increased susceptibility of basal cells may be due to the high expression of these receptors in basally located cells as shown in our scRNA-seq data (Fig. 3A). This receptor distribution may also account for the low *Ctr* infectivity observed by inoculating the hEcCxM models apically, as the barrier function of the stratified epithelium may prevent *Ctr* from reaching the more susceptible intermediate or basal layers.

Although we observed an increase in inclusion sizes at 96 hpi (Fig. 3), inclusions appeared to be restricted to the upper layers, consistent with a previous report in HaCaT cells cultured on inserts [108]. The normal physiological shedding of the uppermost cell layer of EcCx epithelium may also contribute to its resistance to the colonization by *Ctr*. We observed shed cells in both infected and noninfected hEcCxM models, indicating that this process is not solely a consequence of the infection but rather a characteristic feature of the tissue.

The presence of mature and immature inclusions may be due to asynchronous infection related to inoculation without centrifugation and re-infections from ruptured cells at later infection time points. Morphological changes during infection included lesions in early stages, followed by membrane ruffling at later stages. This may indicate a recovery response of the epithelia or indicate cellular stress induced by infection. *Ctr* has also been reported to inhibit apoptosis [109], which could contribute to sustained intracellular survival and delayed host cell turnover.

Interestingly, *Ctr* infection did not induce significant changes in epikine secretion into the basal compartment. This aligns with a previous study using 3D fallopian tube model, which reported minimal secretion of inflammatory cytokines into the basal medium, despite elevated apical secretion of IL10, IL11 and RANTES [110]. Similarly, Bucker et al. [111] observed only modest cytokine secretions in endocervical models. This is in contrast to significant cytokine secretion reported in infected 2D cultures of the upper FRT and immune cells [66,80,112]. These discrepancies may be attributed to model type, epithelial polarization and *Ctr* serovar used. For example, serovar L2 induces a stronger cytokine secretion compared to serovar D [113], which may account for the observed difference in neutrophil migration between *CtrK* and *CtrL2 (*Fig. 4F and G*)*. Furthermore, our data indicates a delayed growth of *CtrK* in the hEcCxM models (Fig. 3), hence stronger neutrophil migration may be induced at later timepoints such as after 48 or 72 h when inclusions were more obvious, compared to 24 h.

The low infection level, the dilution of cytokines in the basal medium, and the absence of immune cells likely contributed to the minimal cytokine detection in the basal medium. Epithelial cells secrete cytokines and chemokines that recruit neutrophil to the sites of infection. The limited influx of neutrophils into the *CtrK* infected hEcCxM may be due to the reduced cytokine secretion. Neutrophils play a role in limiting the spread of *Ctr* infections and also secrete proinflammatory cytokines that may induce the influx of other professional immune cells into the tissue, thus causing further damage to the tissues [114,115].

In conclusion, we have successfully developed and validated an organotypic human ectocervix mucosa model (hEcCxM) that closely recapitulates the transcriptional, cellular and structural architecture of the native ectocervical mucosa. The hEcCxM represents a physiologically relevant platform for investigating mechanisms underlying urogenital tract infections caused by human specific pathogens, as well as non-infectious diseases of the ectocervical mucosa.the models capacity to sustain productive infections long term, combined with its transcriptional fidelity to native tissue, positions it as a valuable tool to evaluate intervention strategies such as novel antimicrobial agents and antibody-based therapeutics derived from vaccines. Furthermore, the model also contribute to the implementation of the 3R principles proposed by Russell and Burch [116] by providing a human-relevant alternative to animal models, especially for human specific pathogen research. The modular design of the platform offers a considerable scope for extension such as the incorporation of the diverse immune cells to investigate epithelial-immune interaction; integration of microbiota and hormones to examine dysbiosis and cycle dependent infection susceptibility respectively. With these extensions, the model can be used to study latent infections, polymicrobial co-infections (e.g. *GC* and *Ctr* co-infection) and ectocervical mucosal immune dynamics governing infection susceptibility in the female genital tract.

## CRediT authorship contribution statement

**Helene Mehling:** Conceptualization, Data curation, Formal analysis, Investigation, Methodology, Writing – original draft, Writing – review & editing. **Claire Rousseau:** Investigation, Methodology, Writing – review & editing. **Tobias Krammer:** Investigation, Methodology, Writing – review & editing. **Alexander M. Leipold:** Data curation, Formal analysis, Visualization, Writing – review & editing. **Aziza Boyny:** Investigation, Writing – review & editing. **Saskia-Laureen Herbert:** Resources, Writing – review & editing. **Christine Wulff:** Resources, Writing – review & editing. **Antoine-Emmanuel Saliba:** Funding acquisition, Methodology, Resources, Supervision, Writing – review & editing. **Lars Dölken:** Funding acquisition, Supervision, Writing – review & editing. **Florian Groeber-Becker:** Funding acquisition, Methodology, Resources, Supervision, Writing – review & editing. **Thomas Rudel:** Conceptualization, Formal analysis, Funding acquisition, Resources, Supervision, Writing – original draft, Writing – review & editing. **David Komla Kessie:** Conceptualization, Data curation, Formal analysis, Investigation, Methodology, Project administration, Supervision, Writing – original draft, Writing – review & editing.

## Declaration of competing interest

The authors declare that they have no known competing financial interests or personal relationships that could have appeared to influence the work reported in this paper.

## Data Availability

Data supporting the findings in this study are available within the paper and its supplementary material. scRNA-seq datasets generated in this study can be assessed at zenodo.org (10.5281/zenodo.19192453). Previously published scRNA-seq datasets that were reanalysed in this study are available from ArrayExpress (accession number: E-MTAB-11948) and National Genomics Data Center (accession numbers: GSE168652 and GSE208653). Only healthy tissues from these datasets were included in the analysis.
