# Supplementary material for: An organotypic ectocervix mucosa model to study host-pathogen interaction of sexually transmitted infections
Source: Mater Today Bio. 2026 Jul 14;39:103454. doi: 10.1016/j.mtbio.2026.103454 (PMC13392605; doi:10.1016/j.mtbio.2026.103454)
Supplement: Multimedia component 1 [file mmc1.docx]

# Supplementary Material

Table S 1: Composition and concentrations ectocervix epithelial growth medium(EcCxGM) adapted from Chumduri et al. [55].

| Component | Supplier | Reference | Final concentration |
| --- | --- | --- | --- |
| Advanced DMEM F12 | ThermoFisher | 12634028 |  |
| 1M HEPES | ThermoFisher | 15630080 | 1 % |
| 100x GlutaMAX | ThermoFisher | 35050061 | 1 % |
| Noggin conditioned medium (HEK293 mNoggin-Fc Cells) | Prof. Dr. Hans Clever, Hubrecht Institute (Gift). | Boonekamp et al, [86] | 10 % |
| B27 | ThermoFisher | 17504-001 | 1 x |
| N2 | ThermoFisher | 17502001 | 1% |
| N-Acetyl-L-Cystein | Sigma Aldrich | A9165-25G | 1.25 mM |
| Nicotinamide | Sigma Aldrich | 72340 | 1 mM |
| Human FGF-10 | ThermoFisher | 100-26-500 | 100 ng/ml |
| Human EGF | ThermoFisher | AF-100-15 | 50 ng/ml |
| Y-27632 Dihydrochloride | Hölzel | 1293823 10mg | 10 µM |
| Forskolin | Bio-techne | 1099/50 | 10 µM |
| Hydrocortison | Sigma Aldrich | H0888-1G | 0.5 µg/ml |
| TGF-b-inhibitor (A83-01) | Bio-techne | 2939/50 | 1 µM |

Table S 2: List of primary antibodies and their dilution used in this study.

| Target | Name (Species) | Supplier | Reference | Dilution used |
| --- | --- | --- | --- | --- |
| KRT5 | Cytokeratin 5 (Rabbit) | Abcam | ab75869 | 1:200 |
| Vimentin | Vimentin (Rabbit) | Abcam | ab92547 | 1:200 |
| ZO-1 | Zona Ocludens-1 (Rabbit) | Proteintech | 21773-1-AP | 1:1000 |
| E-Cadherin | E-Cadherin (Mouse) | BD Biosciences | 610181 | 1:200 |
| TP63 | p63 (Mouse) | Abcam | ab735 | 1:200 |
| KRT13 | Cytokeratin 13(Rabbit) | ThermoFisher | PA5-83165 | 1:1000 |
| MUC1 | Anti-Muc1 antibody | Abcam | Ab109185 | 1:1000 |
| *GC* | Anti-Neisseria gonorrhoeae (Rabbit) | Biomol | N0600-02.1 | 1:1000 |
| MOMP | *Chlamydia trachomatis* MOMP (Mouse) | Biozol | NAC-MAB12270-100 | 1:1000 |
| Actin | Phalloidin-iFluor 405 | Abcam | ab176752 | 1:1000 |
| Actin | Phalloidin-iFluor 647 | Abcam | ab176759 | 1:1000 |
| Actin | Phalloidin AF488 | Abcam | ab176753 | 1:1000 |

Table S 3 Summary of fidelity metrics from integrative analysis

| **Metric** | **Value** |
| --- | --- |
| Shared genes | 19522 |
| Native cells (post-QC) | 15629 |
| Model cells (post-QC) | 7913 |
| Mean label transfer score | 998 |
| Pct cells high-confidence (>0.75) | 99.7 |
| Seurat–SingleR agreement (%) | 96.3 |
| Mean LISI score | 1.003 |
| Jensen-Shannon divergence (composition) | 0.6931 |
| Mean pseudobulk Spearman r | 0.645 |


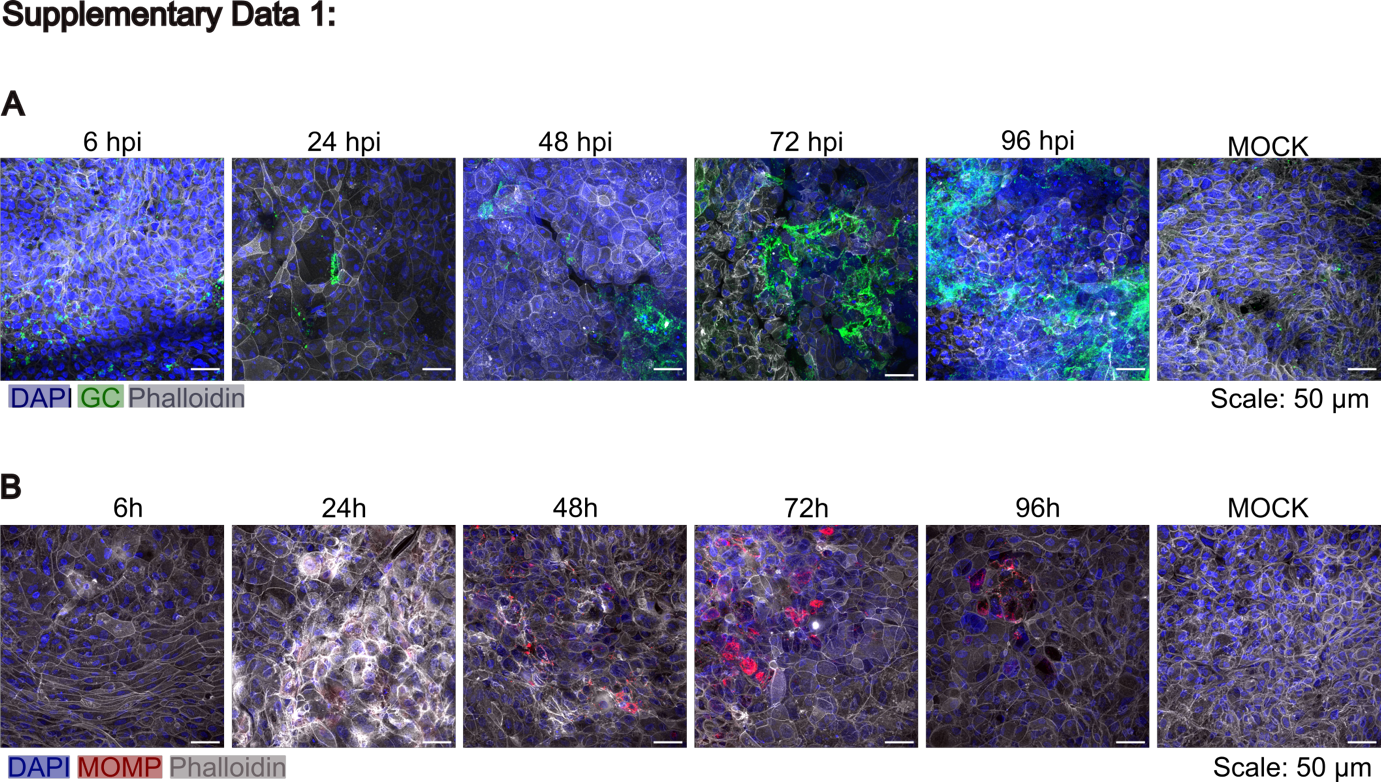


Figure S 1: Representative maximum projection of whole mount immunofluorescent confocal laser scanning microscope images through 100 µm of the hEcCxM models. A: Progress and distribution of *GC(Green)* at different timepoints and Mock *(96 hpi). GC* (green) was stained with anti-GC antibody. B: *Ctr*K inclusion) development at different timepoints post infection. *CtrK*(red) was immunostained with an anti-MOMP antibody. Epithelial cells were stained with phalloidin (gray) and nucleus with DAPI. Scale bar: 50 µm


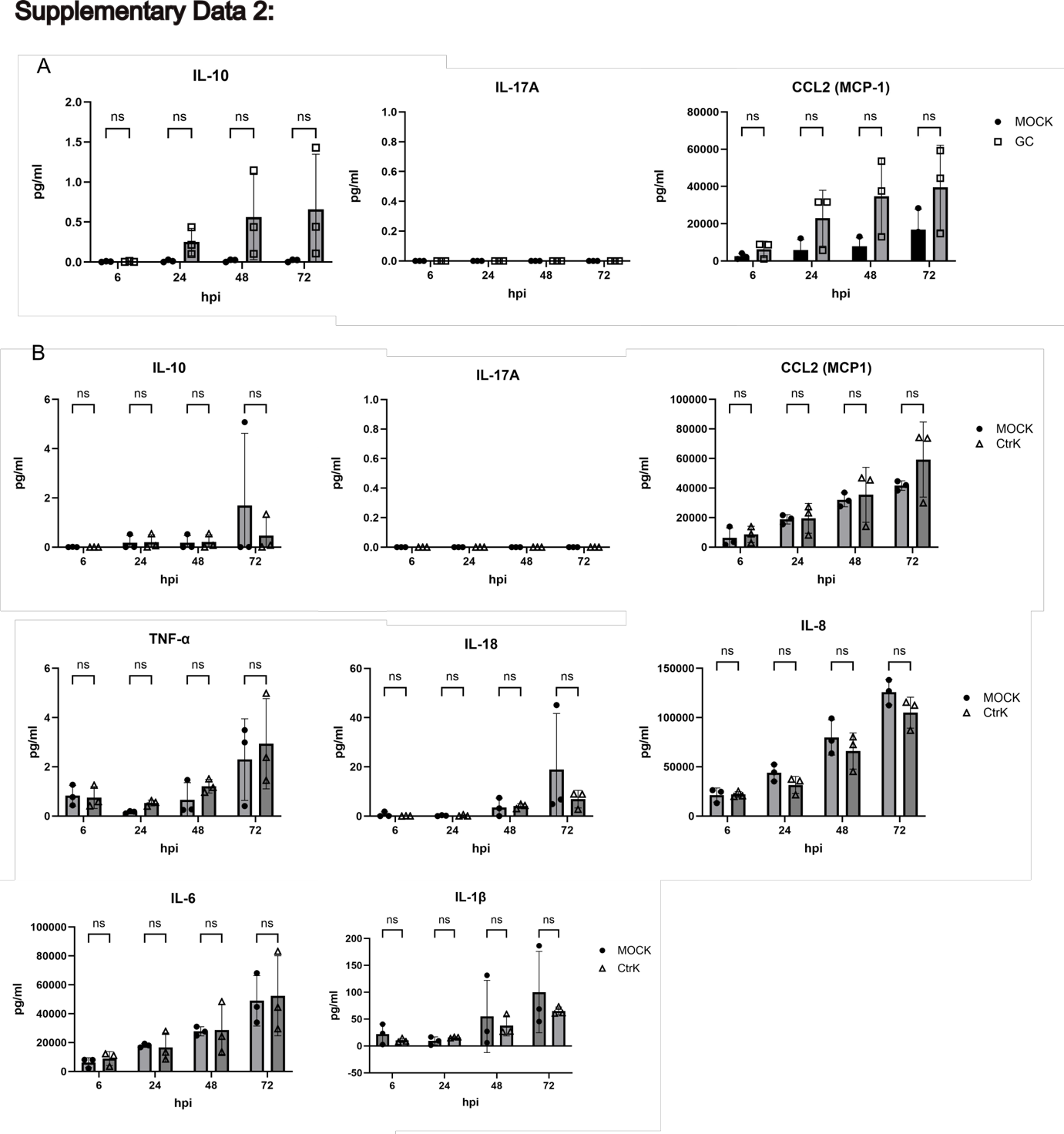


Figure S 2: Cumulative cytokine measurements for infected hEcCxM model over 72h. A: Cytokine results for *GC* infection. IL-10, IL-17A and CCL2 (MCP1) were not significantly increased compared to MOCK. Data represents mean±sd of triplicate of 3 independent experiments from 3 donors (N=3). B: Cytokine results for *CtrK* infection. IL-10, IL-17A, CCL2 (MCP1), TNFa, IL-18 and IL-8 were not significantly increased compared to MOCK. Data represents mean±sd of triplicates of 3 independent experiments from 3 donors (N=3). Significance was tested using 2-way ANOVA and Šídák's multiple comparisons test.


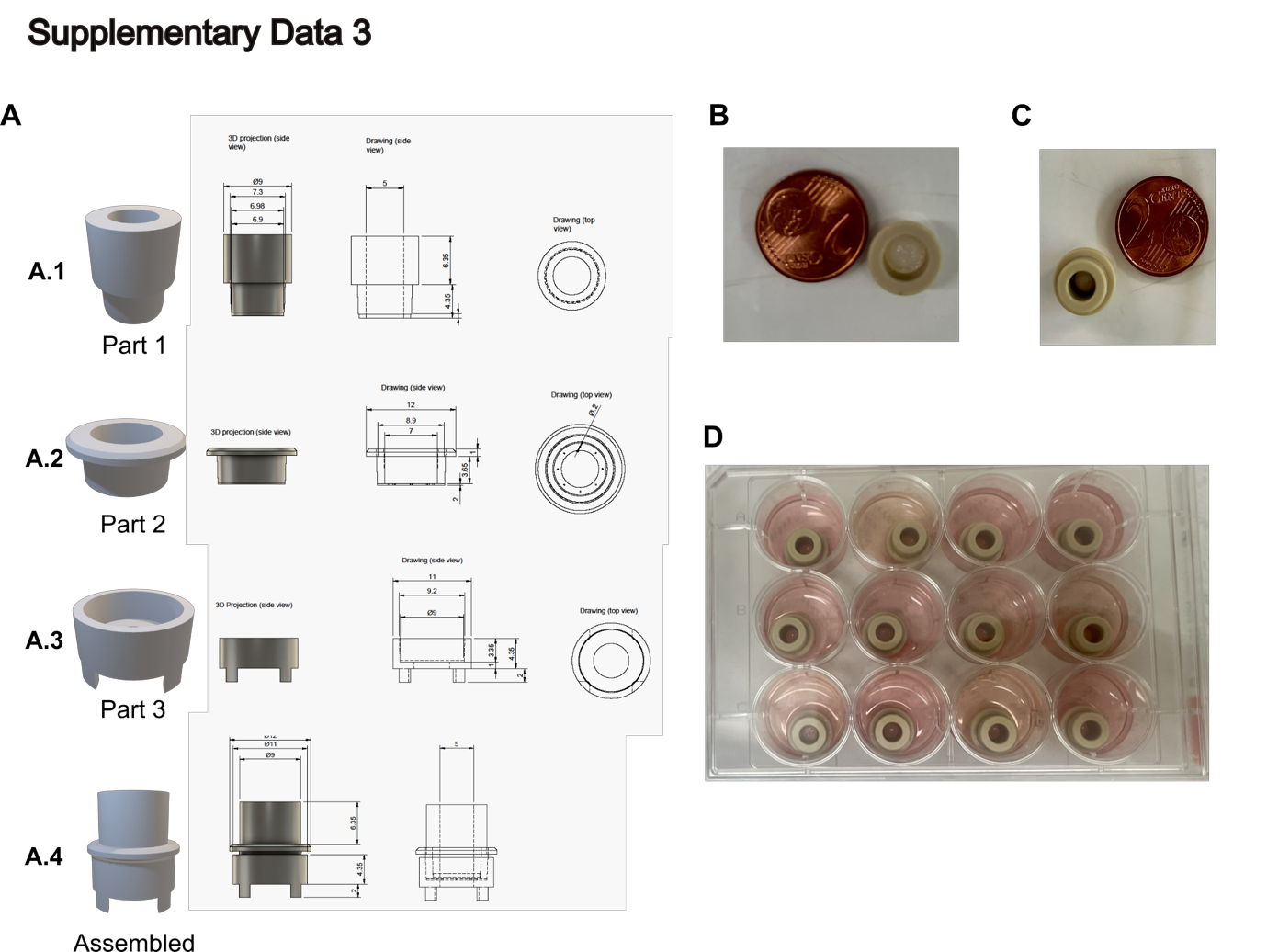


Figure S 3: A: Technical drawing of the custom cell-crowns showing the different parts. Part 2 (A.2) is designed to fit into the compression reactor and provides a chamber with 78,54 mm² surface for 12 mm models and 19,635 mm² for 7 mm models. It holds the plastic collagen (B) during compression and throughout the model culture and infection. After compression, the collagen is locked into place with part 1 (A.1) which fits tightly into part 2. It is hollow to permit seeding of cells on the plastic collagen when the cell crowns are fully assembled. Part 3 (A.3) is the fitted to the base of part 2 and has feet for lifting the model up in the well plate to give access to medium from the basal side. The assembled cell crown (A.4) with the collagen (C) is then placed in well plates (D) and cultured air-liquid-interface until maturity (21-24 days) in a humidified incubator at 37ºC and 5% CO_2_. A 2 cent(euro) coin for scale.


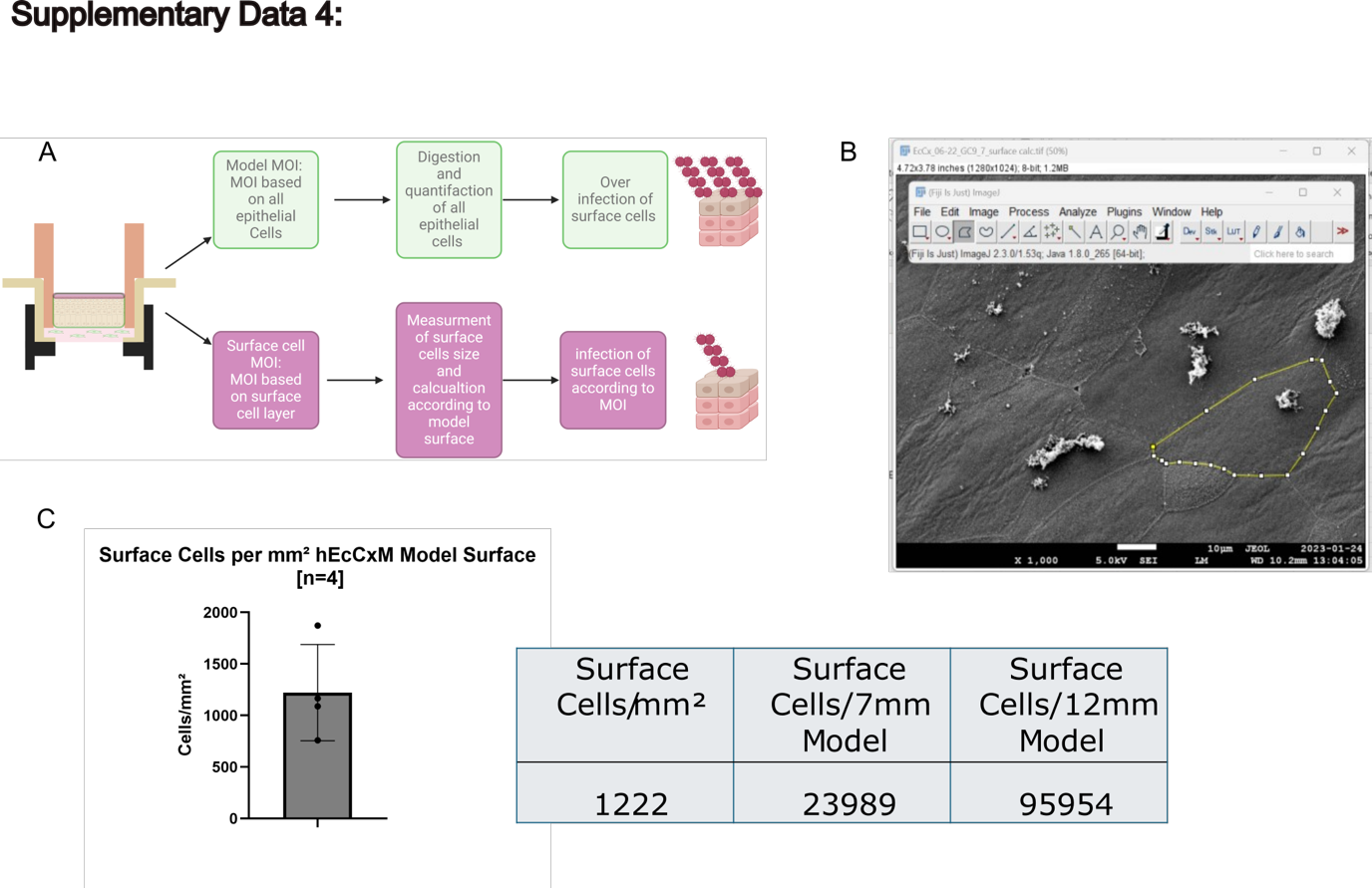


Figure S 4: Establishment of sc_MOI for hEcCxM models. A: Schematic explaining the reason for a surface cell-based MOI in multilayered tissue models compared to a whole model MOI. B: Screenshot showing the surface measurement of the apical layer in the hEcCxM with FIJI. C: Mean surface cell number per mm² on the hEcCxM model with resulting surface cell numbers for 7mm and 12mm models. Data represents mean±sd of triplicates of four independent experiments (n=4).


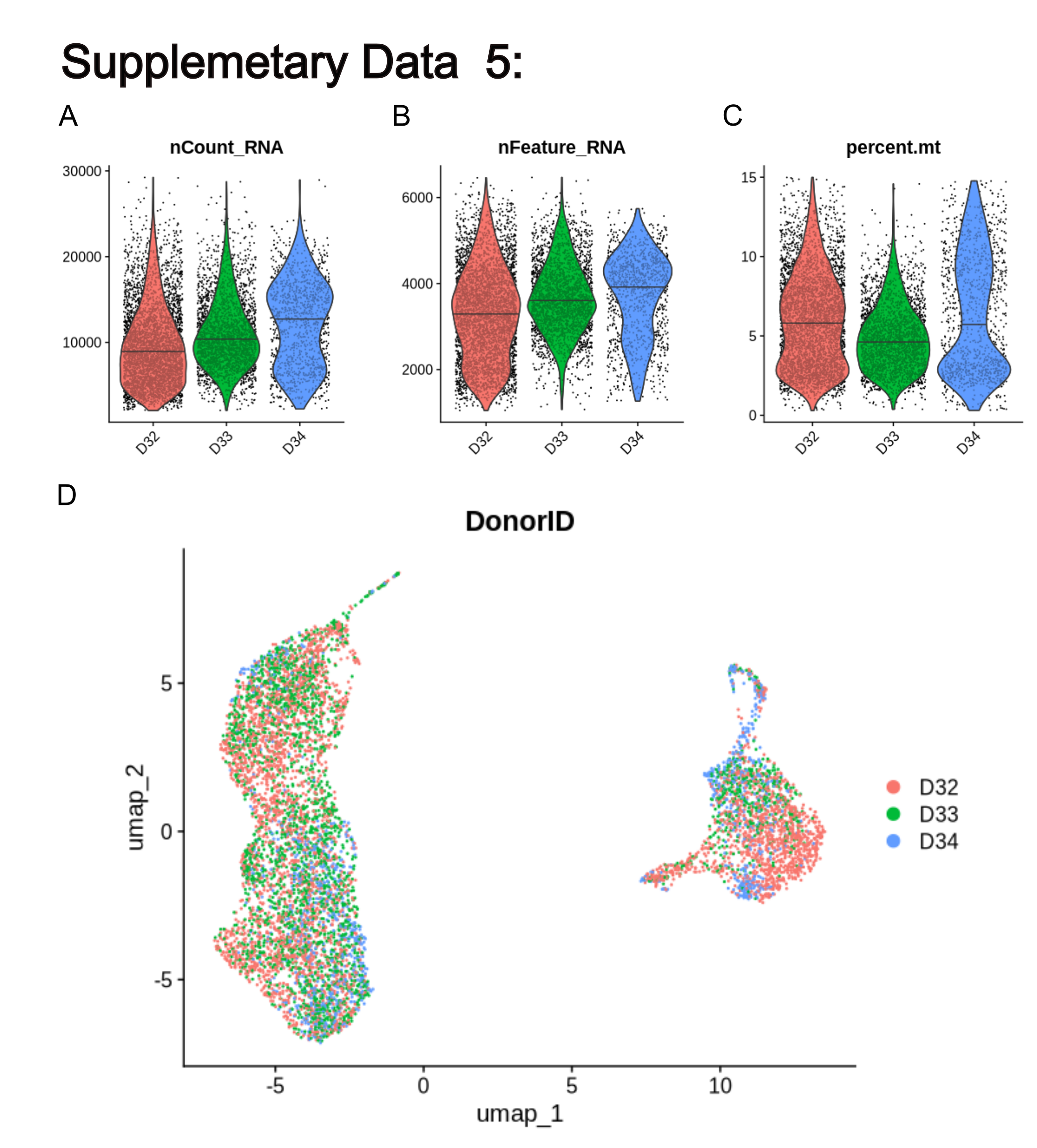


Figure S 5 scRNA-seq quality control data. Violin plots showing fractions of counts (A), features (genes) (B) and fraction of mitochondrial counts(C) per cell from scRNA-seq data. Distribution of cells from human ectocervix mucosa models from three different donors (D) with cells colored by donors.


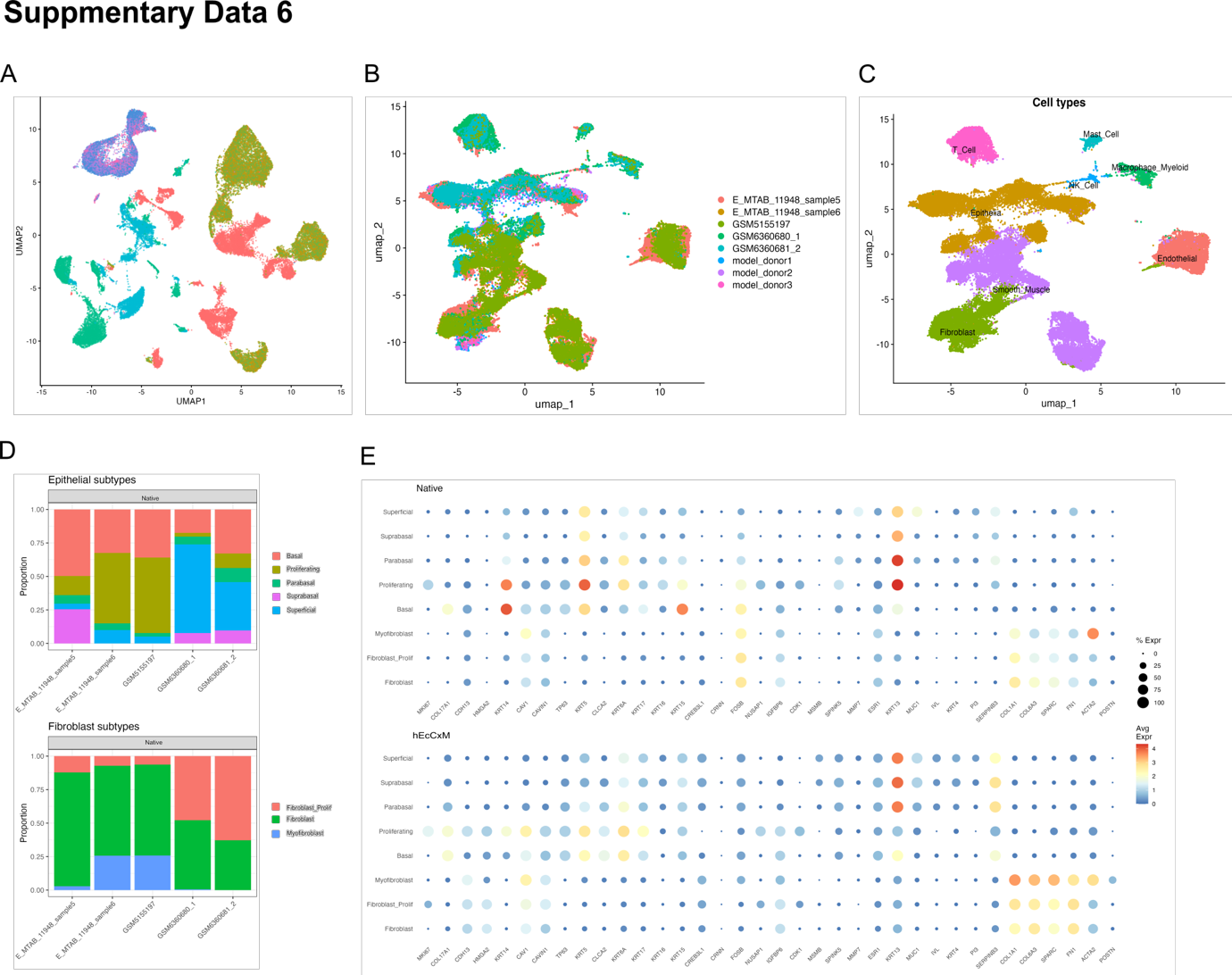


Figure S 6 Integrative analysis of hEcCxM model data and native tissue revealed shared transcriptional signature. A: UMAP projection showing batch-driven clustering and poor cross-dataset alignment of samples from native tissue and hEcCxM datasets before “Harmony” integration. B: UMAP projection of native and hEcCxM model datasets after “Harmony” integration. C: UMAP showing the different cell types from the integration of native and hEcCxM model datasets. D: Proportion of squamous epithelia and fibroblasts subtypes different native samples after subsetting. E: Dotplot showing the expression of typical markers of epithelia and fibroblast subtypes in native and hEcCxM model datasets after integration. Dot size represent proportion of cells expression a particular gene and colour indicates scaled mean expression.
